# Transcriptome analysis of Xenopus orofacial tissues deficient in retinoic acid receptor function

**DOI:** 10.1186/s12864-018-5186-8

**Published:** 2018-11-03

**Authors:** Stacey E. Wahl, Brent H. Wyatt, Stephen D. Turner, Amanda J. G. Dickinson

**Affiliations:** 1 Department of Biology, Virginia Commonwealth University, Richmond, VA USA; 2 Department of Public Health Sciences, University of Virginia School of Medicine, Charlottesville, VA USA

**Keywords:** *Xenopus laevis*, Orofacial development, Cleft palate, Retinoic acid, Transcriptomics

## Abstract

**Background:**

Development of the face and mouth is orchestrated by a large number of transcription factors, signaling pathways and epigenetic regulators. While we know many of these regulators, our understanding of how they interact with each other and implement changes in gene expression during orofacial development is still in its infancy. Therefore, this study focuses on uncovering potential cooperation between transcriptional regulators and one important signaling pathway, retinoic acid, during development of the midface.

**Results:**

Transcriptome analyses was performed on facial tissues deficient for retinoic acid receptor function at two time points in development; early (35 hpf) just after the neural crest migrates and facial tissues are specified and later (60 hpf) when the mouth has formed and facial structures begin to differentiate. Functional and network analyses revealed that retinoic acid signaling could cooperate with novel epigenetic factors and calcium-NFAT signaling during early orofacial development. At the later stage, retinoic acid may work with WNT and BMP and regulate homeobox containing transcription factors. Finally, there is an overlap in genes dysregulated in *Xenopus* embryos with median clefts with human genes associated with similar orofacial defects.

**Conclusions:**

This study uncovers novel signaling pathways required for orofacial development as well as pathways that could interact with retinoic acid signaling during the formation of the face. We show that frog faces are an important tool for studying orofacial development and birth defects.

**Electronic supplementary material:**

The online version of this article (10.1186/s12864-018-5186-8) contains supplementary material, which is available to authorized users.

## Background

The orofacial region, the area between the eyes from the bridge of the nose through the chin, is an integral center for recognition, communication and ingestion. Birth defects affecting this region such as cleft lip and palate are among the most common worldwide. A complete understanding of how the face forms in the embryo is critical to developing methods to prevent these types of birth defects. The face develops through precise coordination of growth and convergence of craniofacial prominences, migration of neural crest cells, and interactions between mesoderm derived tissues and the surrounding ectoderm (reviewed in [1]). We know many of the key players responsible for the orchestration of orofacial development such as WNTs, bone morphogenetic proteins (BMPs), fibroblast growth factors, sonic hedgehog proteins, and retinoic acid (reviewed in [2–6]). However, our understanding of how such signaling pathways interact with each other and implement changes in gene expression is rudimentary. Therefore, this study focuses on uncovering potential cooperation between transcriptional regulators and signaling pathways, in particular retinoic acid, during orofacial development.

Perturbation of retinoic acid (RA) signaling can lead to the development of a cleft lip/palate in humans (reviewed in [7, 8]). Work in animal models has further demonstrated that retinoic acid signals are specifically required for orofacial development [6, 7, 9–13]. In particular, *Xenopus* retinoic acid pathway components are expressed in the developing midface and embryos exposed to an retinoic acid receptor (RAR) antagonist during early orofacial development form a median orofacial cleft [14]. RA ligand binds to a heterodimer of two nuclear receptors most often consisting of RXRs and RARs [15]. These receptors bind to specific enhancer regions in the DNA called retinoic acid response elements. Upon RA binding to RAR/RXR, complexes of coactivators and epigenetic regulators are recruited. These then in turn modify the chromatin structure, allowing the transcriptional machinery to access the DNA and transcription can proceed. Without RA ligand, the receptors are bound by corepressors and repressive epigenetic regulators that stabilize the nucleosome structure so that the DNA is inaccessible to the transcriptional machinery (reviewed in [16, 17]). This balance of RAR activation and repression is integral in regulating gene expression during embryonic development [18].

We now know that RA can modulate the expression of hundreds of genes during development and the expression of such genes can differ widely across developmental events (for examples compare [19–21]). Thus, to gain a more complete understanding of the role of RA during midface development we; 1) examined global gene expression changes in embryos where retinoic acid signals are perturbed and 2) specifically analyzed expression changes in the orofacial tissues during two different phases of its development. By doing so, this work provides a comprehensive picture of how RA is required during orofacial development, independent of its roles in earlier whole body development. Further, we have identified novel signaling and transcriptional regulators that may coordinate with RA during the development of the face. Finally, our work reveals that many of the genes altered in *Xenopus* embryos with a median cleft are also implicated in humans with similar orofacial defects. As a whole, this work furthers our understanding of RA signaling during orofacial development and exhibits that frog faces are an ideal tool for craniofacial research, especially to formulate a more comprehensive understanding of the complex network of signals and transcriptional regulators of this region.

## Results

### Inhibition of retinoic acid signaling during two phases of orofacial development showed overlapping and distinct phenotypes

**Fig. 1:**
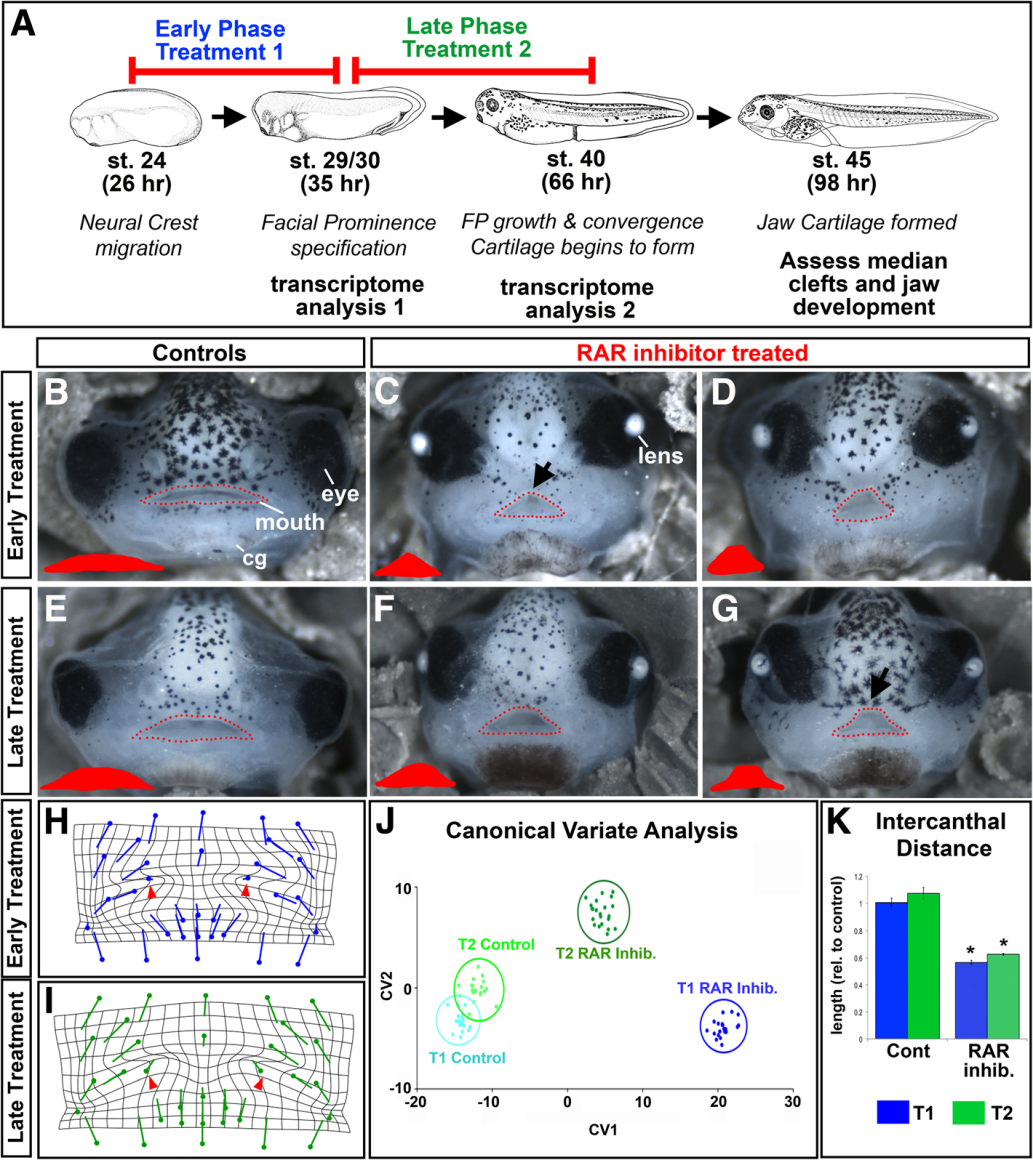
RAR Inhibition during early and late orofacial development. **a** Schematic of the RAR inhibitor treatment paradigm. **b**-**g** Representative images of frontal views of stage 43 (87–92 hpf) embryos. The mouths are outlined in red dots and the shape of the mouth is shown in red in the left corners. b) Embryos treated with 1% DMSO from stage 24 to stage 29/30 (26–35 hpf). **c**,**d** Two different embryos treated from stage 24 to stage 29/30 (26–35 hpf) with 1 uM RAR Inhibitor. Black arrow indicates a triangular shaped mouth **e** Embryos treated with 1% DMSO from stage 29/30 to stage 40 (35–66 hpf). **f**,**g** Two different embryos treated with 1 uM RAR Inhibitor from stage 29/30 to stage 40 (35–66 hpf). Black arrow indicates a mouth shape with a flat dorsal aspect at the midline **h**-**i** Transformation grids exhibiting changes in orofacial landmarks during early (**h**) and late (**i**) RAR Inhibition. Flat end of the vector represents average position of control landmark; closed circle represents average position of landmark in treated embryos. **j** CVA analysis scatter plot. **k** Intercanthal distance relative to control. For all experiments *n* = 22/treatment, 2 biological replicates

To better understand the evolving role of retinoic acid during orofacial development, we perturbed RAR function over two distinct phases. Treatment 1 consisted of RAR antagonist administration during the early phase of facial development, from stage 24–30, (26–35 hpf). At this time the neural crest is migrating and facial prominences are being specified. Treatment 2 consisted of RAR antagonist administration over a later phase from stage 29/30–40, (35–66 hpf; Fig. 1a). During this time the face is growing and facial structures such as jaw cartilage are specified. 100% of the embryos treated with the RAR inhibitor during the early treatment phase developed a median cleft whereas 91% of embryos developed a median cleft with RAR inhibition during the later treatment phase (Fig. 1b-g; *n* = 22/treatment, 2 biological replicates). The median cleft produced by each treatment was assessed by investigating the shape of the mouth. The mouth phenotypes existed as a spectrum, at one end of this spectrum was a median cleft that came to a point, resulting in a triangular shaped mouth (eg Fig. 1c arrow). This phenotype was more prevalent in embryos after treatment 1 (68% of treated embryos). The other shape was where the midline of the dorsal aspect of the mouth was flattened (eg Fig. 1g arrow). This latter phenotype was more common in embryos after treatment 2 (64% of treated embryos).

In order to quantify these differences in the mouth shape, as well as the surrounding midface, we performed a geometric morphometric analysis of embryos treated with the RAR inhibitor. Thirty-three standardized landmarks were used to label the orofacial region for morphometric analysis (Additional file 1: Figure S1B, [14, 22]). Landmark data were aligned by Procrustes fit to eliminate information about size and analyzed using multivariate statistical analyses. First, a discriminant function analysis was utilized to visually assess the changes in landmark position. The results are represented as vector diagrams, such that the flat end of the vector represents the average landmark position in control embryos, and the closed circle represents the average landmark position in the treated embryos. The length of the vectors represents the magnitude of shape change. (Treatment 1 Fig. 1h, Treatment 2 Fig. 1i). These vectors are presented on a transformation grid; warping of the grid highlights regions with the most significant changes. The transformation grids from both the early and late treatments revealed significant shifts that were consistent with a narrowing of the face, especially just above the mouth (Fig. 1h, i). The dramatic warping of the grid in the midface region also exhibited a narrowing of the face. Despite the overall similarity between the changes in landmark position in each treatment phase, there were also important differences. The direction and length of the landmarks just above the mouth were different in embryos after the two different treatments (Fig. 1h, i red arrowheads). This difference could reflect changes in the amount and distribution of primary palate tissue. The landmarks around the mouth were angled differently in the embryos from the two treatment phases, indicative of the spectrum of mouth shape changes that was qualitatively observed (Fig. 1h, i).

Statistical relationships across the RAR antagonist treatment and control groups were visualized simultaneously using a canonical variate analysis (CVA). Data from the CVA is represented as a scatter plot; where each dot represents the average landmark position of a single embryo in a given treatment (Fig. 1j). Procrustes distances (PDs) measure how different one treatment group is from another, with significance indicated by *p*-values. The CVA confirmed the findings of each individual DFA; there were significant differences between the control and RAR antagonist treated groups (Treatment 1 Procrustes distance: 0.2967, *p* < 0.0001, Treatment 2 Procrustes distance: 0.2719, *p* < 0.0001). There was no difference between the two control groups, indicated by the overlap of the circles encompassing average landmark position of the embryos in each condition (Fig. 1j, light blue = control for treatment 1, light green = control for treatment 2;PD = 0.0485, *p* = 0.028). The two different RAR antagonist treatment groups (T1 RAR Inhib, dark blue and T2 RAR Inhib, dark green) appeared separated on the CVA graph (Fig. 1j, PD = 0.0749, *p* = 0.004). However, the Procrustes distance is low and *p*-value is high reflecting only subtle differences in the shape of the orofacial region after early and late RAR antagonist treatments.

Morphometric analysis provided an assessment of the orofacial shape changes but did not address changes in orofacial size. To investigate changes in size, we measured the intercanthal distance, the distance between the eyes. Intercanthal distance was significantly decreased when retinoic acid signaling was inhibited compared to controls during both treatment windows (Fig. 1k, *p* < 0.0001, *n* = 22/treatment with 2 biological replicates). The intercanthal distance was not different between the early and late phase RAR antagonist treated groups (Fig. 1k, *p* = 0.19).

Together our analyses suggested a continuous requirement for retinoic acid during early and late phases of orofacial development. The spectrum of the mouth shape changes shifted from early to late RAR inhibition. This change was reflected in the subtle quantitative shape differences while the size of the face was the same. Further analyses are required to determine how these changes reflect the evolving role of RAR during orofacial tissue development.

### Large scale transcriptome analyses revealed highly altered genes after early and late RAR inhibition

To better understand the underlying mechanisms driving the changes in the shape of the face when embryos have decreased RAR function, we performed two global unbiased expression analyses. For each analysis, the facial region was dissected at the conclusion of the treatment window (Fig. 1a, Additional file 1: Figure S1A). After early phase RAR inhibition, a microarray expression platform assessed relative changes in RNA (Fig. 1a, *n* = 2 biological replicates). An RNA-seq analysis was used to assess relative changes in RNA expression derived from microdissected face tissues after late phase RAR inhibition (reviewed in Fig. 1a, *n* = 3 biological replicates). A random subset of genes were validated by quantitative RT-PCR (Additional file 2: Figure S2); others have been validated in the course of other work in our laboratory [14]. It is important to note that two different methods to evaluate gene expression were utilized, during different years, and therefore the specific genes from each list are not directly comparable.

#### Top genetic alterations after early inhibition of RAR

The most highly altered genes after early RAR inhibitor treatment (Table 1) were first examined. Genes that encode transcription regulators, RNA processing proteins, and calcium signaling mediators, such as CAMKIIG, were among the most decreased transcripts. Genes that were most enriched included those encoding proteins involved in heart and pancreas function and development, suggesting that RA signals inhibit more posterior tissues from developing in the face.

**Table 1:** Most dysregulated genes after early RAR inhibition. The top 20 decreased (left) and top 20 increased (right) genes after RAR inhibition during the early treatment window

Decreased		Increased	
Gene	Full Name	Gene	Full Name
*NCL*	nucleolin	*ANGPTL7*	angiopoietin-like 7
*CKAP2*	cytoskeleton associated protein 2	*SGCB*	sarcoglycan beta
*LUC7L3*	LUC7 like 3 pre-mRNA splicing factor	*PRRC2B*	proline-rich coiled-coil 2B
*CAMK2G*	calcium/calmodulin dependent protein kinase II gamma	*IGFBP1*	insulin-like growth factor binding protein 1
*DNAJB9*	DNAJ heat shock protein family (HSP40) member B9	*TRIM55*	tripartite motif containing 55
*PSAT1*	phosphoserine aminotransferase 1	*NEXN*	nexilin
*TRIM29*	tripartite motif containing 29	*THBS4*	thrombospondin 4
*EFNB2*	ephrin B2	*SH3BGR*	SH3 domain binding glutamate rich protein
*RABEP1*	rabaptin, RAB GTPase binding effector protein 1	*VAMP2*	vesicle associated membrane protein 2
*LARP7*	la ribonucleoprotein domain family member 7	*TLX3*	T-cell leukemia homeobox 3
*USO1*	USO1 vesicle transport factor	*CSNK2A1*	casein kinase 2 alpha 1
*EIF3A*	eukaryotic translation initiation factor 3 subunit A	*ELOB*	elongin B
*RBM25*	RNA binding motif protein 25	*SP3*	sp3 transcription factor
*CHD1*	chromodomain helicase DNA binding protein 1	*SELENOP*	selenoprotein P
*MCPH1*	microcephalin 1	*HECTD1*	HECT domain E3 ubiquitin protein ligase 1
*XPO1*	exportin1	*HOXB2*	homeobox b2
*CIR1*	corepressor interacting with RBPJ, 1	*CAPNS1*	calpain small subunit 1
*LHX8*	LIM homeobox 8	*APOH*	apolipoprotein H
*NRG1*	neuregulin 1	*HABP2*	hyaluronan binding protein 2
*ABCC5*	ATP binding cassette subfamily C member 5	*DHRS3*	dehydrogenase/reductase 3

#### Top genetic alterations after late inhibition of RAR

The most highly altered genes after the late RAR inhibitor treatment (Tables 2 and 3) were also examined. Transcription factors and TGF-beta/BMP family members were among the top decreased genes. In addition, genes in the retinoic acid signaling pathway were also identified in this analysis. Genes with increased expression after late inhibition of RAR included those encoding proteins associated with cells of the eye and the vasculature and heart. Again this result suggests that retinoic acid signals are required to restrict the expansion of surrounding or posterior tissues.

**Table 2:** Most dysregulated genes after late RAR inhibition. The top 20 decreased (left) and top 20 increased (right) genes after RAR inhibition during the early treatment window

Decreased		Increased	
Gene	Full Name	Gene	Full Name
*CYP26A1*	cytochrome P450 family 26, subfamily A, member 1	*FRZB*	frizzle-related protein
*DHRS3*	dehydrogenase/reductase 3	*HBD*	hemoglobin subunit delta
*COCH*	cochlin	*VSX1*	visual system homeobox 1
*CRABP2*	cellular retinoic acid binding protein 2	*CAPN3*	calpain 3
*ALX4*	ALX homeobox 4	*SLC39A8*	solute carrier family 39 member 8
*GATA2*	GATA binding protein 2	*SMOC1*	SPARC related modular calcium binding 1
*SIX2*	SIX homeobox 2	*SIM2*	single-minded family bHLH transcription factor 2
*HIC1*	HIC ZBTB transcriptional repressor 1	*CHODL*	chondrolectin
*RARB*	retinoic acid receptor beta	*DLX2*	distal-less homeobox 2
*SSTR2*	somatostatin receptor 2	*MIP*	major intrinsic protein of lens fiber
*HAS2*	hyaluronan synthase 2	*PMP2*	peripheral myelin protein 2
*TGFBI*	transforming growth factor beta induced	*PCK2*	phosphoenolpyruvate carboxykinase 2, mitochondrial
*CYP26B1*	cytochrome P450 family 26, subfamily B, member 1	*HEMGN*	hemogen
*SFRP1*	secreted frizzled related protein 1	*LHX1*	LIM homeobox 1
*BMP5*	bone morphogenetic protein 5	*IFGBP2*	insulin like growth factor binding protein 2
*BGN*	biglycan	*AIFM3*	apoptosis inducing factor, mitochondria associated 13
*GATA3*	GATA binding protein 3	*ATOH7*	atonal bHLH transcription factor 7
*GH1*	growth hormone 1	*CRYBG1*	crystallin beta-gamma domain containing 1
*DNAJC12*	DnaJ heat shock protein family (Hsp40) member C12	*ERMIN*	ermin
*RHBG*	Rh family B glycoprotein	*CLUL1*	clusterin like 1

**Table 3:** Genes dysregulated after early RAR inhibition associated with transcriptional regulation. Genes shown in Fig. 3 along with their general function (adapted from genecards.org)

*Gene*	Gene Name	General Protein Function
*Decreased*		
*ATRX*	α-thalassemia mental retardation X-linked protein	A SWI/SNF family member protein involved in chromatin remodeling.
*CHD1*	Chromodomain Helicase DNA Binding Protein 1	An ATP-dependent chromatin-remodeling factor.
*CHD2*	Chromodomain Helicase DNA Binding Protein 2	An ATP-dependent chromatin-remodeling factor.
*ESR1*	Estrogen Receptor 1	A nuclear receptor for the estrogen ligand.
*EZH2*	Enhancer Of Zeste 2 Polycomb Repressive Complex 2 Subunit	A polycomb group family member that methylates Lys-9 and Lys-27 of histone H3, leading to transcriptional repression.
*HDAC1*	Histone Deacetylase 1	A histone deacetylase that targets lysine residues on the N-terminal part of the core histones (H2A, H2B, H3 and H4) which is necessary for transcriptional repression.
*SMARCAD1*	SWI/SNF-Related, Matrix-Associated Actin-Dependent Regulator Of Chromatin, Subfamily A, Containing DEAD/H Box 1	A SNF subfamily of helicase proteins that mediates histone H3/H4 deacetylation that is necessary for restoration of heterochromatin organization and propagation of epigenetic patterns following DNA replication.
*SUPT6H*	SPT6 Homolog, Histone Chaperone	A transcription elongation factor which binds to histone H3 and plays a key role in the regulation of transcriptional elongation and mRNA processing.
*SUZ12*	SUZ12 Polycomb Repressive Complex 2 Subunit	A Polycomb group protein that methylates Lys-9 and Lys-27 of histone H3, leading to transcriptional repression.
*UHRF1*	Ubiquitin Like With PHD And Ring Finger Domains 1	A protein that bridges DNA methylation and chromatin modification.
*Increased*		
*AKT1*	AKT Serine/Threonine Kinase 1	A serine/threonine-protein kinase
*BAG6*	BCL2 Associated Athanogene 6	A nuclear protein that forms a complex with E1A binding protein p300 and is required for the acetylation of p53 in response to DNA damage.
*BRD4*	Bromodomain Containing 4	A chromatin reader protein that recognizes and binds acetylated histones and plays a key role in transmission of epigenetic memory across cell divisions and transcription regulation.
*CEBPB*	CCAAT/Enhancer Binding Protein Beta	A transcription factor that contains a basic leucine zipper (bZIP) domain.
*FOS*	Fos Proto-Oncogene, AP-1 Transcription Factor Subunit	A leucine zipper protein that can dimerize JUN and together form part of the AP-1 transcription factor complex.
*GATA3*	GATA Binding Protein 3	A protein that belongs to the GATA family of transcription factors.
*JUN*	Jun Proto-Oncogene, AP-1 Transcription Factor Subunit	A protein that can dimerize FOS and together form part of the AP-1 transcription factor complex.
*MTA2*	Metastasis Associated 1 Family Member 2	A component of NuRD complex which is a deacetylase that is required for remodeling nucleosomes.
*NCOA3*	Nuclear Receptor Coactivator 3	A histone acetyltransferase that recruits p300/CBP as well as CREB binding proteins to form a transcriptional coactivation complex.
*NCOR1*	Nuclear Receptor Corepressor 1	A protein that is associated with thyroid-hormone and retinoic-acid receptors and promotes chromatin condensation and transcriptional repression.
*OTUB1*	OTU Deubiquitinase, Ubiquitin Aldehyde Binding 1	A hydrolase that can specifically remove Lys-48-linked conjugated ubiquitin from proteins and thereby prevents protein degradation.
*PAX7*	Paired Box 7	A member of the paired box (PAX) family of transcription factors.
*PBRM1*	Polybromo 1	A protein necessary for ligand-dependent transcriptional activation by nuclear hormone receptors.
*WBP2*	WW Domain Binding Protein 2	A WW domain binding protein that is a transcriptional coactivator of the estrogen and progesterone receptor.
*Unchanged*		
*SMAD3*	SMAD Family Member 3	An intracellular signal transducer and transcriptional modulator activated by TGF-beta family receptor kinases.

### Functional classification of altered genes after early and late RAR inhibition

We utilized the online analysis software DAVID to categorize the genes perturbed by RAR inhibition into functional processes (version 6.8, NIH [23, 24]. Gene lists were divided into those that were decreased and those that were increased relative to the controls after early and late phase RAR antagonist treatment.

**Fig. 2:**
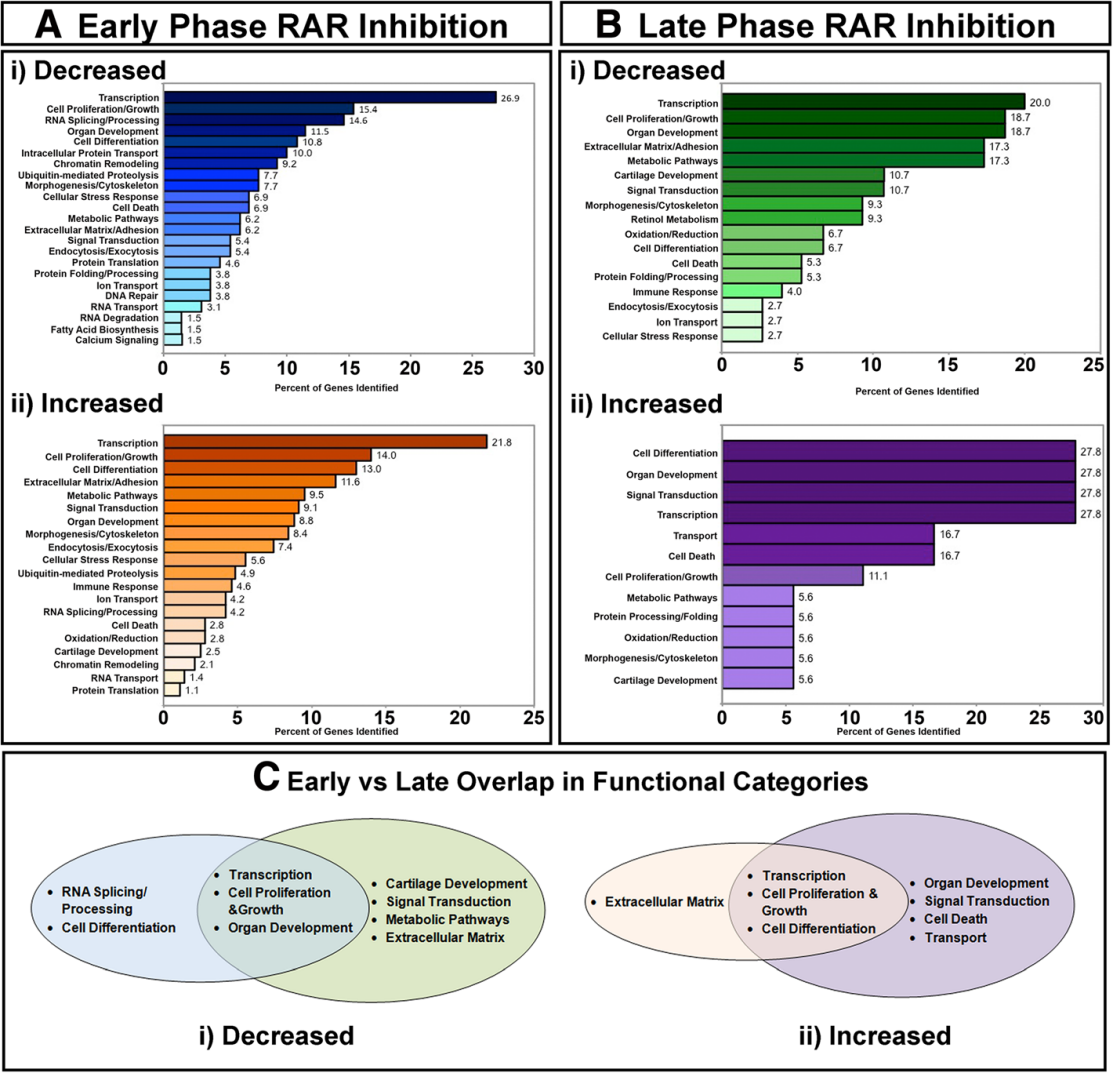
Functional classification of altered genes with RAR inhibition. (**a**). Functional categories of genes decreased (i) or increased (ii) after early RAR inhibition. (**b**). Functional categories of genes decreased (i) or increased (ii) after late RAR inhibition. For all graphs: numbers represent the percent of genes identified. (ci-ii): Venn diagrams representing the overlap between decreased genes (i) and increased genes (ii) in early and late RAR inhibition. Blue and orange represent functional categories altered with early RAR inhibition; green and purple represent functional categories altered with late RAR inhibition

After early RAR antagonist treatment, the top functional categories from depleted gene list were “Transcription” and “Cell Proliferation and Growth” (Fig. 2 Ai). Categories important for the developing embryo “Organ Development” and “Cell Differentiation” were also highly represented in this list. Other functional categories included: “RNA splicing/Processing”, “Intracellular Protein Transport” and “Chromatin Remodeling”. The top functional categories from the enriched gene list after early RAR antagonist treatment were also “Transcription” and “Cell proliferation and Growth” as well as “Cell Differentiation” (Fig. 2 Aii).

After late RAR antagonist treatment, the top functional categories from depleted gene list were “Transcription”, “Cell Proliferation and Growth” and “Organ Development” (Fig. 2 Bi). Other functional categories included: “Extracellular Matrix/Adhesion”, “Metabolic Pathways” and “Cartilage Development”. The top functional categories from the enriched gene list after late RAR antagonist treatment were “Cell Differentiation”, “Organ Development” and “Signal Transduction” (Fig. 2 Bii).

Next, we sought to compare the functional categories identified after each treatment phase. Note, that we comparing the functional groups identified, not the individual genes that were altered. In this analysis we focused on the functional categories representing more than 10% of the genes that were altered after RAR inhibition. This comparison revealed that “Transcription” and “Cell Proliferation/Growth” were both decreased after early and late RAR inhibition (Fig. 2 Ci). This overlap indicated that retinoic acid may be required for these processes throughout orofacial development. Additionally, this finding could offer an explanation for the phenotypic similarities observed after the two treatments (Fig. 1) For example, RAR antagonist treatment during both phases resulted in smaller faces that were not statistically different. Such similar perturbation in the size of the face could be explained by the common effect on genes regulating “Cell Proliferation”. On the other hand, several functional categories did not overlap; this could offer insight into the differences in the phenotype after each treatment. “Cell Death” was a category of genes enriched after the late RAR inhibitor treatment but not the early treatment (Fig. 2 Ci). We could hypothesize that deficient RAR function during the later time points could result in excess apoptosis which could in turn cause a larger gap in the palate region. These analyses provide new information about the potential roles of RA signaling during craniofacial development and allow for the formation of new hypotheses that can be tested in future work.

### Network analysis centered around the functional classification and highly dysregulated genes

While DAVID analysis identified functional categories perturbed after RAR inhibition, it did not provide any information about how signals, pathways, transcription factors and epigenetic modifiers might be integrated in orofacial tissues. Therefore, we utilized Ingenuity Pathway Analysis (version 01–07, Qiagen) to better dissect networks of interacting proteins that were perturbed upon RAR inhibition. Ingenuity Pathway Analysis is a platform that synthesizes literature from human, mouse, and cell culture research to build a database of functional interactions. We utilized this database to build networks between genes that were dysregulated during early or late RAR inhibition, with a focus on two of the top functional categories identified by DAVID analysis Transcription and Organ Development.

#### Networks of genes were identified after early and late RAR inhibition that contained genes from the functional category of “transcription”

Transcription was identified as the largest DAVID functional category of altered genes, after both early and late RAR inhibition. We utilized the previously characterized transcription pathways in Ingenuity to build networks around the most highly altered genes in the transcriptional functional categories after each treatment. For each network, we limited the number of genes to 30 and focused on genes that were altered, minimizing the inclusion of non-affected intermediaries.

##### Transcriptional network altered in early orofacial development contained chromatin regulators and modulators of RAR

**Fig. 3:**
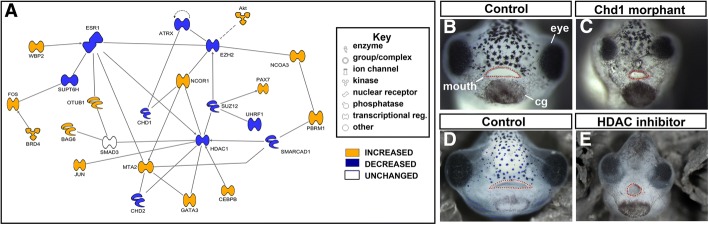
Transcription regulation was altered in early RAR inhibition. **a** Functional network built in IPA software, utilizing DAVID pathway analysis. Blue genes are decreased relative to control; orange genes are increased relative to control. Legend denotes the type of gene product represented in the network. **b**-**c** Representative images of Control (**b**) and CHD1 morphants (**c**). Facial structures are labeled in b. **d**-**e** Representative images of Control (1% DMSO, **d**) and TSA treated embryos (**e**). In all representative images the mouth is outlined in red dots

Among the most highly depleted transcriptional regulators altered the early RAR inhibitor treatment was Chromodomain helicase DNA binding protein 1 (*CHD1*) (see Table 1). *CHD1* belongs to a family of proteins that modify the chromatin and regulate transcription during development [25]. This network linked *CHD1* to several other epigenetic regulators also altered by RAR inhibition, such as *HDAC1, EZH2, ATRX* and *CHD2* (Fig. 3a, Table 3). A subset of the genes from this network encodes proteins that are coactivators or repressors of retinoic acid receptors. For example, *NCOR1, EZH2* (also called *PRC2*) and *HDAC1* encode proteins that belong to complexes that have been shown to repress RAR transcription, while *NCOA* encodes a protein that is a co-activator of retinoic acid [26–31]. Overall, this analysis revealed that the transcriptional regulators that were altered after early RAR inhibition are modulators of chromatin and RAR function.

##### Functional validation: Chromatin modifiers CHD1 and HDACs are required for craniofacial development in *Xenopus* embryos

We sought determine whether the chromatin remodelers identified in our analyses, *CHD1* and *HDAC1* are required for craniofacial development in *Xenopus*. To accomplish this, we decreased the function of these proteins and examined the effects on facial morphology.

A custom designed antisense oligonucleotide morpholino was used to investigate the function of *CHD1* in orofacial development. Embryos were injected at the 1 cell stage with a *CHD1* morpholino or a standard control morpholino; craniofacial phenotypes were assessed at stage 43 (87–92 hpf). *CHD1* morpholino injected embryos had craniofacial defects including midface hypoplasia, misshapen mouth and protruding lenses (*n* = 33, 3 biological replicates, Fig. 3b-c). Further validation and analysis of this protein is provided in another study (Wyatt and Dickinson, in preparation).

To investigate the role of HDACs during early *Xenopus* orofacial development a well-characterized pharmacologic inhibitor, Trichostatin A was utilized [32]. Embryos were exposed to 100 μM Trichostatin A from stage 24–30, (26–35 hpf), transferred to normal culture media after treatment, and assessed for facial phenotype at stage 43 (87–92 hpf *n* = 20/treatment, 3 biological replicates). *HDAC* inhibitor treated embryos had craniofacial phenotypes including: small eyes, lens defects, midfacial hypoplasia, and a small, round mouth when compared to controls (Fig. 3d-e).

In summary, inhibition of either *CHD1* or *HDACs* during the early phase of orofacial development resulted in defects including a narrower midface and smaller mouth, similar to what was observed in embryos with reduced RAR function. Together, these data indicate the possibility that retinoic acid signaling may work with *CHD1* and *HDAC*s during early orofacial development.

##### Transcription network identified with late orofacial development included transcription factors

**Fig. 4:**
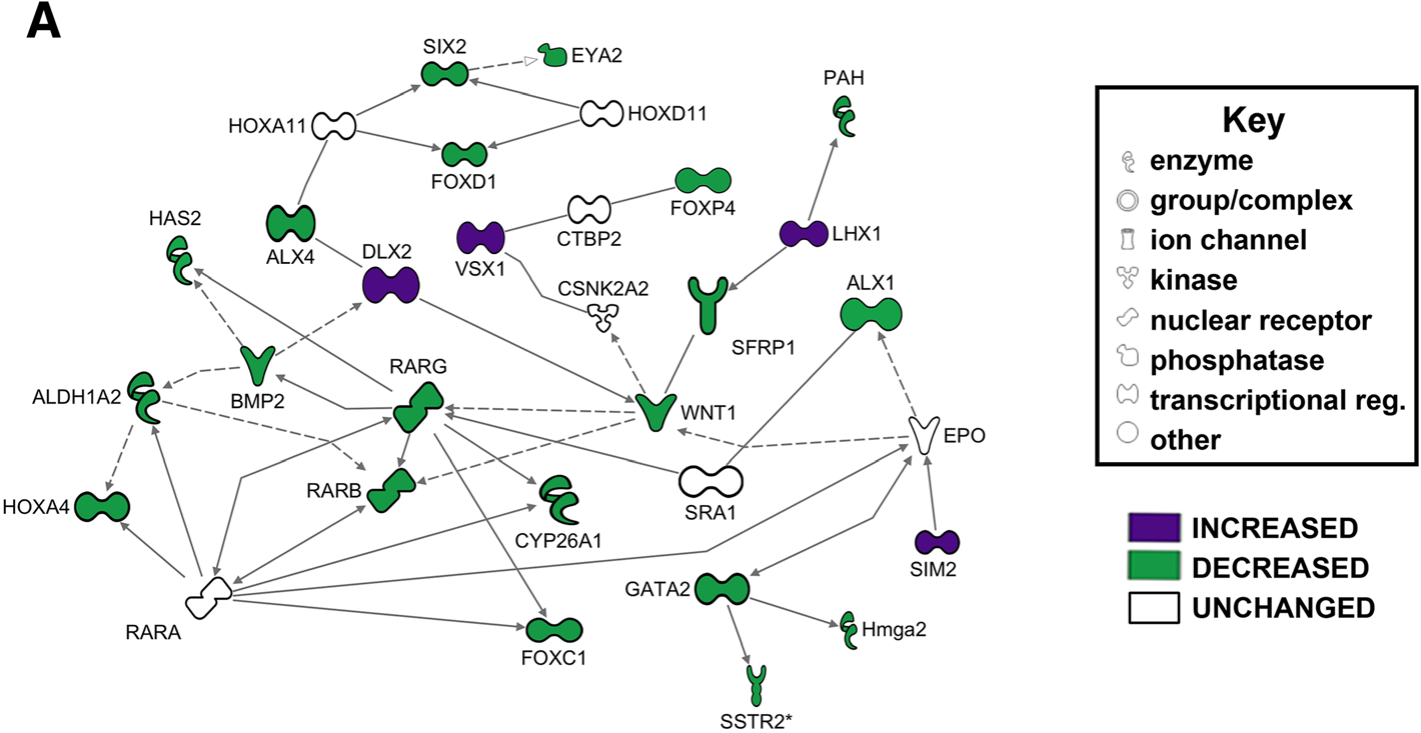
Transcription regulation was largely decreased in late RAR inhibition. Functional network build in IPA software, utilizing IPA and DAVID pathway analysis. Green genes are decreased relative to control; purple genes are increased relative to control. Key denotes the type of gene product represented in the network

Genes identified by DAVID analyses as transcriptional regulators after late RAR inhibition were used to create a network with Ingenuity Pathway Analysis software (Fig. 4 and Table 4). The majority of genes identified in this network were transcription factors. Genes that were depleted included members of the Forkhead (*FOXP4, FOXD1* and *FOXC1*) and Aristaless families (*ALX1* and *ALX4*), as well as *HOXA4*, *SIX2* and *GATA2*. Other transcription factors were enriched in tissues deficient in RAR function, including *DLX2* and *LHX1*. These results reveal that RA signaling is required for the proper expression of transcription factors in the developing face.

**Table 4:** Genes dysregulated after late RAR inhibition associated with transcriptional regulation. Genes shown in Fig. 4 along with their general function (adapted from genecards.org)

*Gene*	Gene Name	General Protein Function
*Decreased*		
*ALDH1A2*	Aldehyde Dehydrogenase 1 Family Member A2	A member of the aldehyde dehydrogenase family of enzymes that catalyzes the conversion of retinaldehyde to retinoic acid, the active derivative of vitamin A.
*ALX1*	ALX Homeobox 1	Unknown in humans, in rodents it is necessary for survival of forebrain mesenchyme and possibly neural tube development.
*ALX4*	ALX Homeobox 4	Homeodomain transcription factor expressed during development of bones, limbs, hair, teeth, and mammary tissue.
*BMP2*	Bone Morphogenetic Protein 2	Secreted ligand of the TGF-beta family that is important in bone and cartilage development.
*CYP26A1*	Cytochrome P450 Family 26 Subfamily A Member 1	Member of the cytochrome P450 superfamily of enzymes. This enzyme catalyzes the oxidation of retinoic acid to render it inactive.
*EYA2*	EYA Transcriptional Coactivator And Phosphatase 2	A member of the eyes absent (EYA) family of proteins that function as a transcriptional coactivator for SIX family proteins.
*FOXC1*	Forkhead Box C1	A member of the forkhead box family of transcription factors important for the regulation of embryonic and ocular development.
*FOXD1*	Forkhead Box D1	A member of the forkhead box family of proteins that are transcription factors, important for kidney, retina, and optic chiasm development.
*FOXP4*	Forkhead Box P4	A member of the forkhead box family of transcription factors that is a transcription repressor.
*GATA2*	GATA Binding Protein 2	Zinc-finger transcription factor that regulates genes involved in development and proliferation of hematopoietic and endocrine cell lineages.
*HAS2*	Hyaluronan Synthase 2	Enzyme involved in the synthesis of hyaluronic acid.
*HMGA2*	High Mobility Group AT-Hook 2	A member of the non-histone chromosomal high mobility group protein family that acts as a transcription regulator.
*HOXA4*	Homeobox A4	A member of the homeobox family of proteins that are transcription factors, important for gene expression, morphogenesis, and differentiation.
*PAH*	Phenylalanine Hydroxylase	Enzyme that catalyzes the rate-limiting step of phenylalanine catabolism.
*RARB*	Retinoic Acid Receptor Beta	Ligand-dependent transcription regulator important for embryonic morphogenesis, cell growth, and differentiation.
*RARG*	Retinoic Acid Receptor Gamma	Ligand-dependent transcription regulator implicated in embryonic growth, limb bud development, and differentiation.
*SFRP1*	Secreted Frizzled Related Protein 1	Soluble modulator of Wnt signaling; important for cell growth and differentiation.
*SIM2*	Single-Minded Family BHLH Transcription Factor 2	Transcription factor that serves as a master regulator of neurogenesis, located within Down’s Syndrome Critical Region.
*SIX2*	SIX Homeobox 2	A member of the sine oculis family of proteins that are transcription factors involved in limb and eye development.
*SSTR2*	Somatostatin Receptor 2	Receptor that mediates the inhibitory effect of somatostatin on hormones and secretory proteins. Also involved in neurodevelopment.
*WNT1*	Wnt Family Member 1	Soluble transcription regulator that binds to frizzled receptors and plays several roles in development.
*Increased*		
*DLX2*	Distal-Less Homeobox 2	Transcription factor expressed in the developing head and limbs; important for the terminal differentiation of neurons and craniofacial patterning.
*LHX1*	LIM Homeobox 1	Transcription factor important for development and differentiation of the neural crest, ectoderm, and urogenital system.
*VSX1*	Visual System Homeobox 1	Transcription factor that regulates opsin gene expression.
*UNCHANGED*		
*CSNK2A2*	Casein Kinase 2 Alpha 2	Protein kinase that phosphorylates acidic C-terminal residues to regulate cell cycle progression, apoptosis, transcription, and viral infection.
*CTBP2*	C-terminal Binding Protein 2	2 alternative transcripts that act as transcriptional repressors and as a component of specialized synapses
*EPO*	Erythropoietin	A hormone synthesized in the kidney that regulates the synthesis of red blood cells in the bone marrow.
*HOXA11*	Homeobox A11	A member of the homeobox family of proteins that are transcription factors, important for gene expression, morphogenesis, and differentiation.
*HOXD11*	Homeobox D11	A member of the homeobox family of proteins that are transcription factors; important for morphogenesis; limb and genital morphogenesis in particular.
*RARA*	Retinoic Acid Receptor Alpha	Ligand-dependent transcription regulator implicated in development, differentiation, apoptosis, and transcription of clock genes.
*SRA1*	Steroid Receptor RNA Activator 1	Transcriptional repressor that binds to non-coding RNAs and has roles in metabolism, adipogenesis, and chromatin organization.

#### Networks of genes were identified after early and late RAR inhibition that contained genes from the functional category of “organ development”

Organ development was a functional category highly represented in the DAVID analysis (Fig. 2). We chose these categories as our next target for Ingenuity Pathway Analysis because genes in this category are integral to developmental processes including facial morphogenesis. Custom networks were built, focusing on the most highly altered genes in the categories of organ development and differentiation. The number of genes in each network was limited to 30 and unaffected intermediaries were minimized.

##### Calcium signaling genes were dysregulated after early RAR inhibition

**Fig. 5:**
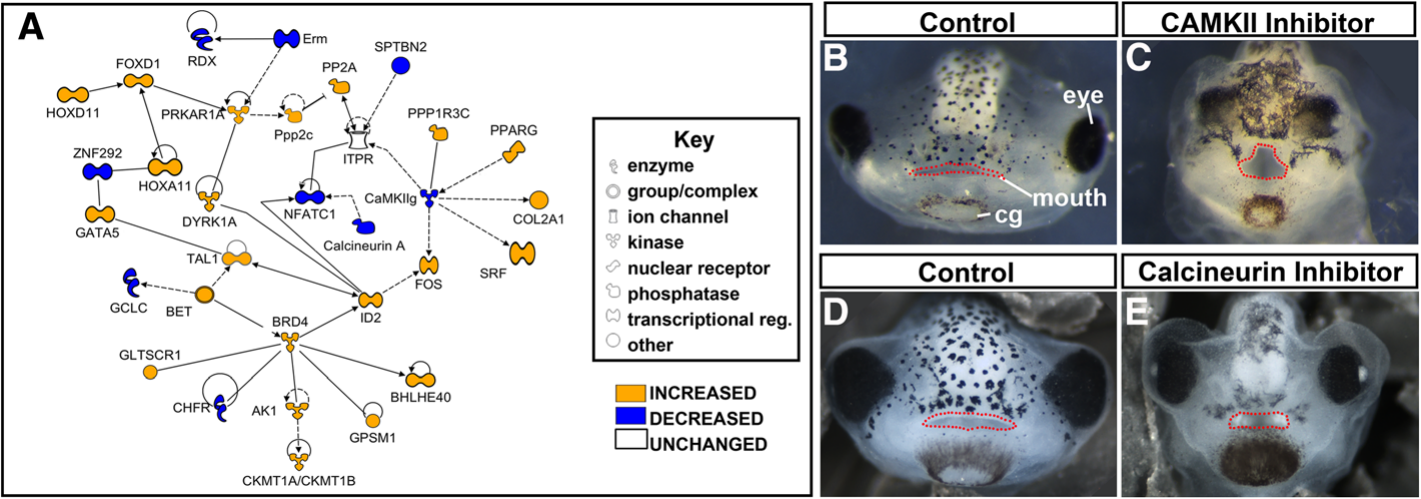
Calcium signaling in Organ Development and Differentiation were altered with early RAR inhibition. **a** Functional network built in IPA software utilizing IPA and DAVID pathway analysis. Blue genes are decreased relative to control; orange genes are increased relative to control. Key denotes type of gene product represented in diagram. **b**-**c** Representative images of control (b, 1% DMSO) and CAMKII inhibitor treated embryos (**c**, 100 μM KN93). **d-e** Representative images of control (1% DMSO, **d**) or calcineurin inhibitor treated embryos (**e**, 100 μM FK506). Mouths are outlined in red dots

Calcium/calmodulin dependent protein kinase 2-isoform gamma (*CAMKIIg*) was highly depleted after early RAR inhibition and was present in the functional category of “Organ Development” in the DAVID analysis. This gene is known to be important for development, which led us to construct an Ingenuity network around *CAMKIIg* (Fig. 5). Predictably, this network identified other proteins involved in calcium signaling such as the calcium channel, Inositol 1,4,5-Trisphosphate Receptor (*ITPR*) and Calcineurin A (*PP3CA*). This network also included *NFAT* and *DYRK1A,* which are misregulated in Trisomy 21 (Fig. 5a and Tables 5).

**Table 5:** Genes dysregulated after early RAR inhibition associated with organ development and differentiation. Genes shown in Fig. 5 along with their general function (adapted from genecards.org)

*Gene*	Gene Name	General Protein Function
*Decreased*		
*CALCINEURIN A (PPP3CA)*	Protein phosphatase 3 catalytic subunit alpha	Calcium-dependent phosphatase that is activated by calmodulin and involved in CAMKII signaling
*CAMKIIG*	Calcium/Calmodulin Dependent Protein Kinase II Gamma	Serine/threonine protein kinase, activated by calcium and/or calmodulin with several roles in development, particularly in neurodevelopment
*CHFR*	Checkpoint With Forkhead And Ring Finger Domains	E3 ubiquitin-protein ligase required for the maintenance of the G2/M checkpoint, may play a key role in cell cycle progression
*ERM (ETV5)*	ETS variant 5	Transcription factor that binds to DNA with consensus sequence 5-GGAA-3, may be disregulated in cancer.
*GCLC*	Glutamate-Cysteine Ligase, Catalytic Subunit	Glutamate-cysteine ligase, rate-limiting enzyme of glutathione synthesis.
*NFACTC1*	Nuclear Factor Of Activated T-Cells 1	A component of the NFAT DNA-binding complex. Members of this family play central roles in inducible gene transcription during immune response.
*RDX*	Radixin	Cytoskeletal protein that important in linking actin to the plasma membrane.
*SPTBN2*	Spectrin Beta, Non-Erythrocytic 2	Component of spectrin, important in securing glutamate transporter EEAT4 to the plasma membrane and regulating glutamatergic signaling.
*ZNF292*	Zinc Finger Protein 292	Growth hormone-dependent transcription regulator.
*Increased*		
*AK1*	Adenylate Kinase 1	Catalyzes the reversible transfer of the terminal phosphate group between ATP and AMP
*BET*	Bromodomain containing, BET	Family of genes that contain 110 amino acid domains that recognize acetylated lysine residues and translate the acetylation signal into various phenotypes depending on the family member’s location and expression.
*BHLHE40*	Basic Helix-Loop-Helix Family Member E40	Basic helix-loop-helix protein expressed in various tissues and is believed to be involved in the control of circadian rhythm and cell differentiation.
*BRD4*	Bromodomain Containing 4	Protein with a conserved sequence motif involved in chromatin targeting and binds acetylated lysine residues.
*CKMT1A/CKMT1B*	Creatine Kinase, Mitochondrial 1A, Creatine Kinase, Mitochondrial 1B	Serine/threonine kinases that play a central role in energy transduction in tissues with large, fluctuating energy demands, such as skeletal muscle, heart, brain and spermatozoa.
*COL2A1*	Collagen Type II Alpha 1 Chain	Alpha-1 chain of type II collagen, a fibrillar collagen found in cartilage
*DYRK1A*	Dual Specificity Tyrosine Phosphorylation Regulated Kinase 1A	Located on the Down’s Syndrome Critical Region, this gene product is involved in cell survival, proliferation, quiescence, and differentiation.
*FOS*	Fos Proto-Oncogene, AP-1 Transcription Factor Subunit	Leucine zipper protein that dimerizes with proteins of the JUN family to form the transcription factor AP-1; regulates cell proliferation, differentiation, and transformation.
*FOXD1*	Forkhead Box D1	Member of the forkhead family of transcription factors, functions in the differentiation of nephron progenitors and the development of the retina.
*GATA5*	GATA Binding Protein 5	Transcription factor containing 2 GATA-type zinc fingers, involved in establishment of cardiac smooth muscle diversity.
*GLTSCR1 (BICRA)*	BRD4-interacting chromatin domain remodeling complex associated protein	Plays a role in BRD4-mediated transcription.
*GPSM1*	G Protein Signaling Modulator 1	A receptor-independent activator of G protein signaling, regulates spindle orientation and cortical neuronal cell fate. May also be involved in macroautophagy in the intestine.
*HOXA11*	Homeobox A11	Transcription factor that provides cells with specific positional identities on the anterior-posterior axis.
*HOXD11*	Homeobox D11	Transcription factor that provides cells with specific positional identities on the anterior-posterior axis.
*ID2*	Inhibitor Of DNA Binding 2	Basic helix-loop-helix transcription regulator that binds to other HLH transcription regulators and inhibits their activity; important for glial differentiation and chondrocyte development.
*PP2A (PPP2CA)*	Protein phosphatase 2 catalytic subunit alpha	Catalytic subunit of serine/threonine phosphatase involved in the negative regulation of cell growth.
*PPARG*	Peroxisome Proliferator Activated Receptor Gamma	Forms heterodimers with retinoid X receptors (RXRs) to regulate transcription.
*PPP1R3C*	Protein Phosphatase 1 Regulatory Subunit 3C	Carbohydrate binding protein subunit of the protein phosphatase 1 (PP1) complex. Involved in the maintenance of glycogen levels by activating/inhibiting synthesis and breakdown enzymes.
*PPP2C*	Protein Phosphatase 2 Catalytic Subunit	Catalytic subunit of serine/threonine phosphatase involved in the negative regulation of cell growth.
*PRKAR1A*	Protein Kinase cAMP-Dependent Type I Regulatory Subunit Alpha	Regulatory subunit of the cAMP-dependent protein kinases involved in cAMP signaling in cells including GPCR signal transduction.
*SRF*	Serum Response Factor	Ubiquitous transcription factor that stimulates proliferation and differentiation.
*TAL1*	TAL BHLH Transcription Factor 1, Erthyroid Differentiation Factor	Basic helix-loop-helix transcription factor that plays a role in hematopoietic and erthyroid differentiation.
*UNCHANGED*		
*ITPR*	Inositol 1,4,5 triphosphate receptor family	Family of membrane glycoproteins that act as calcium channels to regulate several cellular processes including proliferation, learning, and memory.

**Table 6:** Genes dysregulated after late RAR inhibition associated with organ development and differentiation. Genes shown in Fig. 6 along with their general function (adapted from genecards.org)

*Gene*	Gene Name	General Protein Function
*Decreased*		
*ALX1*	Alx Homeobox 1	Unknown in humans, in rodents it is necessary for survival of forebrain mesenchyme and possibly neural tube development.
*BGN*	Biglycan	Small, leucine-rich proteoglycan that plays a role in bone growth, muscle development, and collagen fibril assembly.
*BMP2*	Bone Morphogenetic Protein 2	Secreted ligand of the TGF-beta family that is important in bone and cartilage development.
*BMP5*	Bone Morphogenetic Protein 5	TGF-beta family ligand that binds to BMP receptors and activates Smad signaling. Important for bone and cartilage development.
*COL9A1*	Collagen Type IX Alpha 1 Chain	Essential for assembly of type IX collagen a minor component of hyaline cartilage and found in tissues containing type II collagen which forms cartilage
*COL9A2*	Collagen Type IX Alpha 2 Chain	Essential for assembly of type IX collagen a minor component of hyaline cartilage and found in tissues containing type II collagen which forms cartilage
*COL9A3*	Collagen Type IX Alpha 3 Chain	Essential for assembly of type IX collagen a minor component of hyaline cartilage and found in tissues containing type II collagen which forms cartilage
*HMGA2*	High Mobility Group AT-Hook 2	A member of the non-histone chromosomal high mobility group protein family that acts as a transcription regulator.
*RARB*	Retinoic Acid Receptor Beta	Ligand-dependent transcription regulator important for embryonic morphogenesis, cell growth, and differentiation.
*RARG*	Retinoic Acid Receptor Gamma	Ligand-dependent transcription regulator implicated in embryonic growth, limb bud development, and differentiation.
*SIX2*	SIX Homeobox 2	A member of the sine oculis family of proteins that are transcription factors involved in limb and eye development.
*TGFB1*	Transforming Growth Factor Beta 1	Ligand for TGF-beta superfamily of signaling molecules, activates Smad signaling to regulate cellular proliferation and differentiation.
*WNT1*	Wnt Family Member 1	Soluble transcription regulator that binds to frizzled receptors and plays several roles in development.
*Increased*		
*DLX2*	Distal-Less Homeobox 2	Transcription factor expressed in the developing head and limbs; important for the terminal differentiation of neurons and craniofacial patterning.
*FRZB*	Frizzled Related Protein	Modulator of Wnt signaling important for the regulation of bone development.
*SLC39A8*	Solute Carrier Family 39 Member 8	Manganese and zinc influx transporter.
*COL2A1*	Collagen Type II Alpha 1 Chain	Fibrillar collagen found in cartilage and the vitreous of the eye, essential for normal embryonic development of the skeleton
*Unchanged*		
*ACAN*	Aggrecan	Integral extracellular matrix proteoglycan in cartilaginous tissue
*CHRDL2*	Chordin Like 2	Chordin family member that interacts with TGF-beta signaling and may play a role in myoblast and osteoblast differentiation.
*SIX5*	SIX Homeobox 5	Transcription factor involved in the regulation of organogenesis.
*SNAI1*	Snail Family Transcriptional Repressor 1	Zinc finger transcriptional repressor that downregulates the expression of ectodermal genes in the mesoderm.
*TGFB2*	Transforming Growth Factor Beta 2	Ligand for TGF-beta superfamily of signaling molecules, activates Smad signaling.

##### Functional validation: CAMKII and CALCINEURIN are required for craniofacial development in *Xenopus* embryos

We validated the findings of this network by examining altered levels of two proteins identified in the Ingenuity analysis, *CAMKIIg* and *Calcineurin A* (also called *PPP3CA*). To determine if these proteins were required during the early orofacial development phase, pharmacological inhibitors were utilized to diminish their function.

The antagonist KN93 was used to disrupt CaMKII function. KN93 prevents the association between calmodulin and CaMKII and has been widely used in experimental models, including zebrafish [33]. Embryos were exposed to 100 μM KN93 from stage 24–30 (26–35 hpf), the same time window as our early RAR inhibitor treatment (reviewed in Fig. 1a, *n* = 20, 2 biological replicates). After treatment, embryos were raised until stage 43 (87–92 hpf) and then facial phenotypes were assessed. KN93 treated embryos had a narrower midface, close-set eyes and protruding lenses (Fig. 5b-c). The shape of the mouth resembled those treated with the RAR inhibitor with a median cleft that either came to a point (not shown), or formed a gap (Fig. 5c).

The antagonist FK506, a previously validated inhibitor, was used to inhibit Calcineurin during early orofacial development in *Xenopus* [34]. Embryos were treated with 100 μM of FK506 from stage 24–35 (26–50 hpf, n = 20, 2 biological replicates) and facial morphology was assessed at stage 43 (87–92 hpf). FK506 treated embryos had a narrower midface, protruding lenses, and a square shape mouth (Fig. 5d-e).

In summary, inhibition of either CaMKII or Calcineurin during the early phase of orofacial development resulted in defects similar to RAR inhibitor treated *Xenopus* embryos. These results suggest that RA signaling may interact with calcium signaling during orofacial development.

##### BMP and WNT family members were dysregulated after late RAR inhibition

**Fig. 6:**
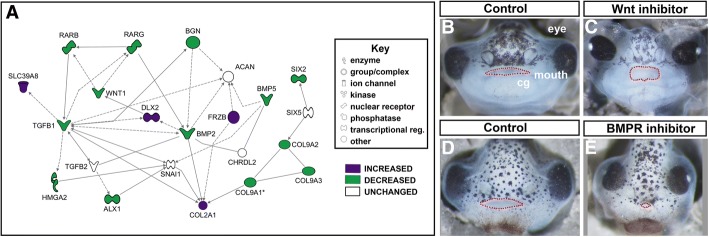
Wnt and BMP signaling were altered with late RAR inhibition. **a** Functional network built in IPA software utilizing IPA and DAVID functional analyses. Green genes are decreased and purple genes are increased relative to control. Key denotes type of gene product represented in diagram. **b**-**c** Representative images of control (**b**, 1% DMSO) and Wnt inhibitor treated embryos (**c**, IWR-1 10 μM). **d**-**e** Representative images of control (**d**, 1% DMSO) and BMPR Inhibitor (**e**, LDN193189, 100 μM). Mouths are outlined in red dots

*BMP5* and the Wnt antagonist *FRZB* were among the most highly changed genes after late RAR inhibition. These genes were also identified in the functional category of “Organ Development”. An Ingenuity network was constructed around these genes and identified additional members of the WNT and BMP pathways, including *WNT1, BMP7*, as well as *TGFB1* a close relative of BMP. This network also connected genes associated with cartilage (*COL9A*, and *BGN*) and transcription factors *ALX1, SIX2* and *DLX2* (Fig. 6a, Table 6).

##### BMP and WNT signals are required for craniofacial development in *Xenopus*

We functionally tested whether BMP and WNTs are required for craniofacial development during the late orofacial development phase. To accomplish this, we utilized pharmacological inhibitors that could specifically test the role of these morphogens during this phase of orofacial development, without perturbing earlier events in development.

To disrupt BMP signaling, Type I BMP receptors, which are activated by BMP2 and BMP5 were blocked utilizing an established pharmacological antagonist [35, 36]. Embryos were treated with 100 μM of LDN193189 from stage 30–40 (35–66 hpf, *n* = 20, 3 biological replicates, Fig. 6d-e). Facial characteristics were assessed at stage 43 (87–92 hpf). The midface appeared narrow, and the mouth was both smaller and rounder (Fig. 6e). These results demonstrate that BMP is required during late phase orofacial development in *Xenopus*.

The pharmacological inhibitor IWR-1, was next utilized to block the WNT-induced accumulation of β-catenin through stabilization of the destruction complex member AXIN2 [37–40]. Embryos were treated with 10 μM IWR-1 from stage 29/30–40 (Fig. 6b-c, 35–66 hpf, *n* = 20 from 3 biological replicates). At stage 43 (87–92 hpf) facial characteristics were assessed. IWR-1 treated embryos showed lens and eye defects, a narrower face and smaller misshapen mouth (Fig. 6c).

In summary, these experiments revealed that both BMP and Wnt are required over a very specific period of orofacial development.

### Genes dysregulated in Xenopus after RAR inhibitor treatments are associated with human craniofacial birth defects

**Fig. 7:**
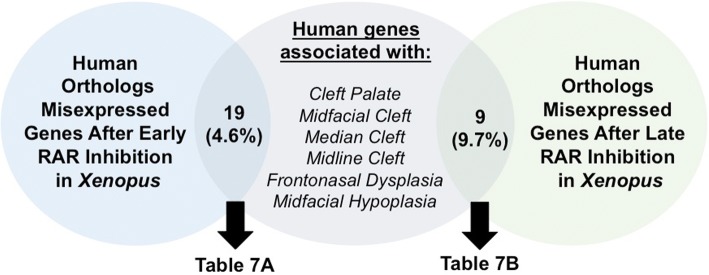
Overlap of altered gene expression in RAR deficient face and human orofacial defects. Venn diagram exhibiting the overlap among genes altered in our treatment paradigm in *Xenopus* and human median orofacial defects. Blue circle: total number of *Xenopus* genes with human orthologs (415) that were significantly altered with early RAR inhibition. Green circle: total number of *Xenopus* genes with human orthologs (93) that were significantly altered with late RAR inhibition. Grey circle: Human genes associated with one of the six craniofacial defects listed (325 total). Overlap of circles denotes genes that appeared in both lists

In *Xenopus*, RAR inhibition during specific windows of development result in median clefts in the primary palate [14]. While this phenotype resembles the human median cleft, it remained to be determined if the phenotypes had similar genetic origins. We explored this question by investigating whether genes altered in our *Xenopus* cleft phenotype were associated with a human craniofacial defect. Using the Online Mendelian Inheritance of Man database (OMIM.org), we searched for 6 keyword terms that applied to symptoms of a defect in the midface region (listed in Fig. 7). 406 human genes were associated with these keywords (Additional file 3: Table S1). We then compared the list of human genes to the significantly altered genes after both early and late RAR inhibition (Additional file 3: Table S1). Of the 415 genes that were significantly altered after early RAR inhibition, 19 were associated with a median orofacial defect (4.6%, Fig. 7, Table 7A). After late RAR inhibition, 9 of the 93 genes that were significantly altered were also associated with a median orofacial defect (9.7%, Fig. 7, Table 7B). These results suggest that retinoic acid could also regulate a number of genes that can cause midface birth defects in humans.

**Table 7:** Genes associated with human medial craniofacial defects that were dysregulated with RAR inhibition in *X. laevis*. A-B: Human orthologs of *Xenopus* genes significantly altered with early RAR inhibition (A) or late RAR inhibition (B) that were associated with a craniofacial defect (compiled with the use of OMIM.org)

*Gene*	Associated Disease with Median Orofacial Defect	Disease OMIM Number
A: Genes with Altered Expression After Early RAR Inhibition		
*ALDOA*	GLYCOGEN STORAGE DISEASE XII	611,881
*ATRX*	ALPHA-THALASSEMIA/MENTAL RETARDATION SYNDROME, X-LINKED	301,040
	MENTAL RETARDATION-HYPOTONIC FACIES SYNDROME, X-LINKED	309,580
*BMP2*	BRACHYDACTYLY, TYPE A2	112,600
*CHRNG*	MULTIPLE PTERYGIUM SYNDROME, ESCOBAR VARIANT	265,000
*COL11A1*	MARSHALL SYNDROME	154,780
	FIBROCHONDROGENESIS 1	228,520
	STICKLER SYNDROME, TYPE II	604,841
*COL2A1*	STICKLER SYNDROME, TYPE 1	108,300
	KNIEST DYSPLASIA	156,550
	SPONDYLOEPIPHYSEAL DYSPLASIA CONGENITA	183,900
*COL9A2*	STICKLER SYNDROME, TYPE IV	614,284
*EFNB1*	CRANIOFRONTONASAL SYNDROME	304,110
*GJB2*	VOHWINKEL SYNDROME	124,500
*KLHL41*	NEMALINE MYOPATHY 9	615,731
	NEMALINE MYOPATHY 3	161,800
*MAFB*	OROFACIAL CLEFT 1	119,530
*MYH3*	ARTHROGRYPOSIS, DISTAL, TYPE 2A	193,700
*PITX1*	CLUBFOOT, CONGENITAL, WITH OR WITHOUT DEFICIENCY OF LONG BONES AND/OR MIRROR-IMAGE POLYDACTYLY	119,800
*RAPSN*	FETAL AKINESIA DEFORMATION SEQUENCE	208,150
*SMC3*	CORNELIA DE LANGE SYNDROME 3	610,759
*TRAPPC9*	MENTAL RETARDATION, AUTOSOMAL RECESSIVE 13	613,192
B: Genes with Altered Expression After Late RAR Inhibition		
*ALX1*	FRONTONASAL DYSPLASIA 3	613,456
*ALX4*	PARIETAL FORAMINA 2	609,597
	FRONTONASAL DYSPLASIA 2	613,451
*BMP2*	BRACHYDACTYLY, TYPE A2	112,600
*COL9A2*	STICKLER SYNDROME, TYPE 1	108,300
*CYP26C1*	FOCAL FACIAL DERMAL DYSPLASIA 4	614,974
*DLX2*	AXENFELD-RIEGER SYNDROME, TYPE 1	180,500
*GATA3*	HYPOPARATHYROIDISM, SENSORINEURAL DEAFNESS, AND RENAL DISEASE	146,255
*HIC1*	LISSENCEPHALY 1	607,432
	MILLER-DIEKER LISSENCEPHALY SYNDROME	247,200
*RARB*	MICROPHTALMIA, SYNDROMIC 12	615,524
*SMOC1*	MICROPHTHLAMIA WITH LIMB ANOMALIES	206,920

## Discussion

Here we combine transcriptional expression analysis, functional validation and comparison with human databases to provide a global view of the effects of retinoic acid perturbation and clefting in a model vertebrate.

### Retinoic acid requirement during early orofacial development

We defined “early” orofacial development to include the time when cranial neural crest cells migrate and facial prominences are specified. Also during this period, the first steps in embryonic mouth occur and the cranial placodes are formed.

#### Epigenetic regulation

Generally during development, retinoic acid is an important morphogen that directs gene expression that is in turn necessary for specifying cell fates. Therefore, it would not be surprising that RA is also required for maintaining the expression of transcriptional machinery, epigenetic factors and cofactors required for gene expression during orofacial specification. Consistent with this hypothesis, we identified a network of genes dysregulated in facial tissues deficient for RAR function, which included chromatin readers and remodelers. For example, two Chromodomain helicase DNA-binding (CHD), *CHD1* and *CHD2*, were decreased in *Xenopus* embryos exposed to a RAR antagonist. Further, functional validation revealed that *CHD1* is required for orofacial development in *Xenopus*. The CHD family of enzymes belong to the SNF2 superfamily of ATP-dependent chromatin remodelers and are associated with active transcription (reviewed in [41]). Complete loss of *CHD1* in mice is devastating; embryos die before craniofacial development can occur [42]. Other CHD family members, specifically *CHD7* and *CHD8* are essential for craniofacial development in mice and humans [43–45]. For example, *CHD7* is associated with CHARGE syndrome, which can include craniofacial defects such as a small mouth, and cleft lip and/or cleft palate and are the result of problems in neural crest development [44, 45] In addition to *CHD* family members, our analysis uncovered other chromatin readers and remodelers including *ATRX* [46], *BRD4* (MGI) and *MTA2* [47] that are also associated with craniofacial birth defects. Current work in the lab is investigating the role of *CHD1* and other epigenetic regulators during *Xenopus* craniofacial development.

RARƴ and other retinoic acid pathway members are expressed in the face throughout both early and later orofacial development in *Xenopus*. We might expect, therefore, that during early orofacial development it is necessary to maintain and perpetuate retinoic acid signaling. To accomplish this, RAR function could be critical for the expression of its partners. Indeed our analysis identified a network that included chromatin remodelers and cofactors involved in RAR receptor mediated transcriptional regulation. Traditionally, upon retinoic acid binding, histone modifying complexes are recruited to target genes to promote their transcription. Notably, we determined that one such histone modifying complex member, *NCOA3*, was dysregulated in the face tissues of *Xenopus* embryos treated with and RAR antagonist. On the other hand, in the absence of retinoic acid, members of several repressive complexes are recruited to target genes to promote transcriptional repression. Several such complex members including *HDAC1 EZH2, SMARCAD1, SUZ12,* and *NCOR1*, were dysregulated in embryos with deficient RAR function. Functional validation demonstrated a distinct role for HDACs during the early phase of orofacial development, which is supported by work in other model vertebrates [48–50].

Together our results suggest that RAR function is required for maintaining the proper expression levels of a number of epigenetic regulators necessary for retinoic acid mediated gene expression. A role for retinoic acid in regulating DNA methylation, histone modification, and miRNA expression has also been demonstrated during development, stem cell maintenance and in cancer (reviewed in [51]). Thus, while a role for retinoic acid in regulating epigenetic factors is not unexpected, a better understanding of the coordination of RAR and chromatin remodelers will provide a more complete view of how this important morphogen gradient is utilized during the development of the face.

#### Calcium-NFAT pathway

Network analysis of genes dysregulated in the orofacial tissues after early RAR inhibition identified several connected genes encoding proteins associated with calcium signaling such as the calcium channel *ITPR, CAMKIIg* and *Calcineurin A*. Functional validation demonstrated that *CaMKII* and *Calcineurin* are indeed required during early orofacial development in *Xenopus*. Calcium levels in the cell mediate a multitude of processes and events during development (reviewed in [52]). The importance of proper calcium regulation insures that there are several modulators of calcium levels, depending on the tissue type and process. For example, calcium can be released from internal stores upon binding of inositol triphosphate to a receptor that acts as a calcium channel (ITPR). Calcium levels then modulate calcium sensitive kinases such as Ca^2+^/calmodulin-dependent protein kinases (CaMK) and phosphatases, such as calcineurin. These enzymes regulate the activity of a wide range of signaling and transcription factors in the embryo (see [52]). One such transcription factor, identified in our network analysis, is nuclear factor of activated T cells (*NFAT*) [53]. Calcineurin dephosphorylates this protein, which results in its translocation to the nucleus. Once in the nucleus, NFAT can activate the transcription of many different genes that in turn influences diverse cellular functions including diversification of neural crest cells and craniofacial development [54–57] . In *Xenopus, nfatc3* is expressed in tissues consistent with the neural crest during early orofacial development (Xenbase). Another regulator of NFAT activity that was identified in our network analysis of genes dysregulated after RAR inhibition was *DYRK1A*. This protein phosphorylates NFAT triggering its movement into the cytoplasm and thus preventing its transcriptional activity. Haploinsufficiency of *DYRK1A* results in craniofacial defects that can include cleft lip/palate in humans [58, 59]. Further, *DYRK1A* is located on the Down’s Syndrome Critical Region of chromosome 21 and has been linked to the characteristic facial features associated with this disorder [60]. Moreover, the craniofacial features associated with increased *DYRK1A* dosage closely resemble those of *NFAT* and *Calcineurin* loss of function in mice [57]. The *Calcineurin-NFAT-DYRK1A* axis has also been shown to interact with *CAMKIIg*. CAMKII inhibits NFAT nuclear translocation by phosphorylation and inhibition of Calcineurin in cardiac myocytes [61]. While we know that CAMKII is integral to embryonic development, including signaling in cilia [62–64], a role in craniofacial development has never been demonstrated. Therefore, a novel avenue for future work will be to investigate the roles of CAMKII, Calcium signaling and NFAT regulation and how these interact with retinoic acid signaling during orofacial development.

In summary, we demonstrate that retinoic acid is required for the expression of epigenetic factors and members of Calcium-NFAT signaling which in turn could influence events such as neural crest migration and specification of the facial prominences during early orofacial development.

### The requirement for retinoic acid during late orofacial development

We defined late orofacial development to include a time when facial prominences grow and converge as well as the differentiation of the facial tissues. During this time the embryonic mouth is formed and jaw cartilage and muscle begin to form.

#### WNT/beta-catenin and BMP

Our work suggests that during the later period of orofacial development, retinoic acid signaling cooperates with well-known regulators of craniofacial development, *WNT* and TGF-beta family members including *BMP*. Ingenuity pathway analysis identified a network of dysregulated genes connecting *WNT1*, *FRZB, BMP2* and *BMP5*. Further, functional validation using pharmacological WNT and BMPR antagonists over this late phase of orofacial development resulted in *Xenopus* embryos with defects in the midface. Specifically, embryos treated with a WNT antagonist developed a large round embryonic mouth and narrower midface. This is consistent with previous work *Xenopus that* demonstrated that overexpression of Wnt inhibitors *frzb-1*, *crescent* and *dkk* similarly resulted in a larger mouths but narrower faces [65]. Together, work in various vertebrate models indicate that Wnt/beta-catenin inhibitors define the region that will form the embryonic mouth while Wnt/beta-catenin signals are required in the surrounding facial prominences to maintain proliferative regions and direct jaw cartilage development [66–70]. Thus, disruption of the balance of WNT activation and inhibition can alter the relative facial dimensions in the developing embryo [71]. Next we determined that *Xenopus* embryos exposed to a BMPR antagonist over the late phase of orofacial development resulted in both a smaller mouth and midface. Consistently, mice deficient for *BMP4* during orofacial development have decreased facial growth and reductions in the jaw skeleton (reviewed in [72]{Shuman, 2007 #3380} [73]. In humans, mutations in members of either WNT or BMP pathways are associated with craniofacial defects [74–78] Thus, a role for WNT/beta-catenin and BMP signaling during orofacial development of *Xenopus* is not surprising [79].

Interactions between WNT, BMP and retinoic acid have also been previously demonstrated in craniofacial development in model organisms and humans. For example, exogenous retinoic acid suppresses canonical Wnt in the developing palate [80, 81]. Retinoic acid interacts with BMPs to determine the identity of the frontonasal mass and maxillary prominences in developing chick embryos [82, 83]. Additionally, teratogenic levels of retinoic acid and BMP signaling in humans contribute to the severity of holoprosencephaly, a disorder with characteristic midline facial defects [84]. Thus, our study demonstrates that *Xenopus,* like other vertebrates, likely requires the coordination of retinoic acid, WNT and BMP signals to direct orofacial development.

#### Transcription factor network

Retinoic acid signaling is well known for its role in regulating the expression of transcription factors, especially those with a homeobox {Langston, 1994 #3084} [18, 85]. Therefore, we were not surprised to uncover a network of homeobox containing transcription factors disrupted in orofacial tissues with deficient RAR function. Several of these transcription factors (*ALX1, ALX4, FOXC, FOXD1* and *SIX2*) are required for craniofacial [86–91]. Moreover, at least of subset of such transcription factors could be direct targets of retinoic acid signaling. The promoter regions of two genes in our analysis, *FOXP4* and *HOXA4,* were bound by RAR in embryonic stem cells. These genes were also activated upon exogenous retinoic acid application [92]. Additional experiments would be necessary to determine whether a subset of the transcription factors identified in this work are indeed direct targets of retinoic acid signaling during orofacial development. Regardless, these results present the possibility that retinoic acid signals coordinate either directly or indirectly a host of transcription factors critical for the development and differentiation of the facial tissues.

### XENOPUS as a tool for understanding human orofacial birth defects

Twenty-six genes were dysregulated in orofacial tissues after RAR inhibition are associated with specific birth defects affecting the midface (Table 7, Fig. 7). Humans with these defects present with orofacial abnormalities that share features observed in *Xenopus* embryos deficient in retinoic acid signaling. For example, birth defects including Stickler Syndrome and Frontonasal Dysplasia include midline or median clefts, a hallmark of RAR deficiency in *Xenopus*. Therefore, in humans just as in Xenopus, retinoic acid signals may coordinate the expression of many of the genes required for the formation of the midface and associated tissue types such as cartilage and bone. This could mean that in cases where human midface defects are a result of changes in the dosage of RA-regulated genes, RAR agonists and vitamin A supplementation could potentially serve as future therapies. Our work demonstrates that *Xenopus* could be an ideal organism in which to test the therapeutic potential of retinoic acid analogs for preventing or ameliorating craniofacial birth defects.

## Conclusions

This study utilized *Xenopus laevis* to study retinoic acid-mediated mechanisms of orofacial development. Through this work, we confirmed the efficacy and usefulness of *Xenopus* as a craniofacial model. Further, we determined signaling pathways and networks affected by RAR inhibition and provided functional validation of members of those networks in orofacial development. We identified several genes that are altered with RAR inhibition in *Xenopus* that correspond to genes altered in human disorders. This work provides evidence for how RA regulates orofacial development and identifies several targets for further research into the etiology of facial development in *Xenopus,* which may provide insight for human facial development.

## Methods

### Obtaining *Xenopus* embryos

*Xenopus laevis* adults were created in our breeding colony and purchased from Nasco. All procedures were approved by the VCU Institutional Animal Care and Use Committee (IACUC protocol number 5 AD20261). Embryos were collected using standard procedures [93] and were staged according to Nieuwkoop and Faber [94]. Briefly, eggs were collected in high salt 1X Modified Barth’s Saline (MBS, pH 7.8) from at least two different females and in vitro fertilized using sperm obtained from one male. Embryos were cultured in 0.1X MBS, refreshed daily, until used for experiments. Embryos were housed in a 23 °C or 15 °C incubator (Torrey Pines Scientific, Cat. No. IN30). After the experiments were completed all embryos were given a lethal dose of anesthetic (10% tricaine).

### Pharmacological inhibitor treatments and antisense Morpholinos

Two periods of development were chosen for our analysis based on craniofacial developmental events. An early period or phase, when the neural crest is migrating and facial prominences are specified. A later phase, when the face is growing and the jaw muscle and cartilage just begin to form. At the beginning of each phase (early = stage 24 or late = 30), embryos were arrayed in 60 mm culture dishes containing 10 ml of 0.1X MBS with the desired chemical and DMSO (final concentration of DMSO was 1%). Chemicals used for analysis were: 1 μM RAR antagonist (BMS453, 1 mM stock solution in DMSO, Tocris, Cat. No. 3409), 100 μM CaMKII antagonist (KN93, 5 mM stock solution in water, Sigma-Aldrich Cat. No. 422711-M), 125 μM HDAC inhibitor (Trichostatin A, 5 mM stock solution in DMSO, Sigma-Aldrich, Cat. No T8852), 25 μM Dyrk1a inhibitor (INDY, 100 mM stock solution in DMSO, Tocris, Cat. No. 4997), 40 μM Wnt1 inhibitor (10 mM stock solution in DMSO, Sigma-Aldrich, Cat. No. I0161), and 25 μM BMPR1 Inhibitor (LDN-193189, 10 mM stock solution dissolved in water, Calbiochem, Cat. No. 509882). During pharmacological treatments, embryos were maintained at 23 °C in an Echotherm incubator (Torrey Pines Scientific, Cat. No. IN30). Antisense morpholinos (MOs) with fluorescein tags were purchased from Genetools. 60 ng of a splice blocking Chd1 MO (CTAGTCACTTTACCTCTGCTCCATC) was injected into the 1 cell stage. The equivalent standard control morpholino was used as a control in this experiment. In all experiments at least 40 embryos were treated in 2 biological replicates.

### Intercanthal distance measurements and Morphometrics

Embryos were fixed in 4% paraformaldehyde (PFA) overnight at 4 °C. A sterile disposable No. 15 scalpel (VWR, cat. no: 82029–856) and Dumont No. 5 forceps (Fisher, cat. no: NC9404145) were used to make two cuts to isolate the head: first- at the posterior end of the gut and then second caudal to the gills. Isolated heads were mounted in small depressions in a clay-lined dish containing Phosphate Buffered Saline with 0.1% Tween (PBT) [95]. The faces were imaged using a Discovery.V8 stereoscope fitted with an Axiovision digital camera (Zeiss). AxioVision40 V.4.8.1.0 software (Zeiss) was used to measure the *intercanthal distance,* the distance between the eyes. Statistical Analysis: Student’s t-tests assuming unequal variance between each treatment group and corresponding control were performed to determine statistical relationships. Error bars representing standard error were calculated in Excel.

Morphometrics analysis was performed as described previously with some modifications [95]. A total of 33 landmarks were placed on tadpole faces using the Pointpicker plugin of ImageJ (NIH, Additional file 1: Figure S1B). Landmarks 1–5 were placed on the midline of the face between the top of the cement gland and the top of the eyes. Landmarks 6–17 outlined each eye. Landmarks 18–27 completed the outline of the midface region from the center of the eyes to the top of the cement gland, eliminating variation due to forebrain or cement gland size. Landmarks 28–33 outlined the mouth opening. Landmark coordinates were analyzed in MorphoJ 1.05f with Procrustes superimposition to remove information concerning scale, size, position, and orientation [96]. Statistical Analysis: To study statistical relationships among the treatment paradigms and corresponding controls, a canonical variate analysis (CVA) of Procrustes fit landmark data was performed and visualized as a bivariate plot. Discriminate function analysis (DFA) was also performed to examine statistical relationships between each treatment and its respective control. These were represented as transformation grids, in which the flat end of the vector represented average landmark position in control embryos, and the round end of the vector represented average landmark position in RAR-inhibitor exposed embryos. Statistical relationships are represented by *p*-values and all p-values that are less than 0.0001 are shown as *p* < 0.0001 as designated by MorphoJ.

### Orofacial microdissections and RNA extraction

The orofacial region of control and RAR-inhibitor treated embryos were isolated by manual dissection at 35 or 66 hpf. Briefly, the embryo was placed in a chilled dish of 0.1X MBS (15C) and the head was isolated. The target area surrounding the stomodeum or mouth (Additional file 1: Figure S1A), excluding most of the brain and eyes was removed using a disposable scalpel with a 15 T blade Sklar Instruments, Cat. No 06–3120). Dissected tissues were added to TriReagent (Sigma, Cat. No. T9424) and frozen at − 80 °C until RNA Isolation. 1 Biological replicate consisted of approximately 100 embryos from 2 to 3 pooled experiments. RNA extraction was conducted using the TriReagent protocol and lithium chloride precipitation (Ambion, AM9480). RNA quantity was assessed by Quawell UV-Vis spectrophotometer Q5000 and accompanying software.

### Microarray and RNAseq

A microarray expression analysis was performed by the genome core at Virginia Commonwealth University (VCU) using the *Xenopus laevis* Genome 2.0 Array GeneChip containing probes to over 32,000 probe sets which represented more than 29,000 *X. laevis* transcripts (# 901214, Affymetrix). The VCU core performed RNA quality analysis, cDNA construction, and hybridization to the GeneChips, expression and statistical analysis. Statistical Analysis: Statistics and relative gene expression was performed with the Affymetrix statistical expression algorithm provided in the gene Chip Operating Software package. Data was compiled into an Excel file for further analysis in the Dickinson lab. *X laevis* genes that were significantly altered by 1.75 fold or greater (415 unique genes) were converted to the human ortholog for further functional and network analyses. Genetic similarity was determined to be > 90% identity and similarity. The files generated by this analysis are publically available at NCBI Gene Expression Omnibus [97] accession number GSE116819. Genes identified and used in this analysis are provided in table S2 (Additional file 4).

RNAseq was performed by Hudson Alpha Institute for Biotechnology (Huntsville, AL) where a HiSeq v4 PE100 250 mil reads 50 GB max output with Teir 1 analysis performed. The resultant data was analyzed by Dr. Stephen Turner and colleagues at the Bioinformatics Core at the University of Virginia (Charlottesville, VA). Reads were aligned using *Xenopus laevis* reference genome and STAR alignment software, and then counted using featureCounts software in the Subread package (http://www.xenbase.org/, RRID:SCR_003280) [98–100]. Statistical Analysis: The DESeq2 Bioconductor package in the R statistical computing environment to normalize count data, estimate dispersion, and fit a negative binomial model for each gene [101–103]. R Code available upon request. Data was then exported to Excel for further analysis in the Dickinson lab. *Xenopus* genes that were significantly altered (*p* < 0.05) were converted to the human ortholog for functional and network analyses (93 unique genes). Genetic similarity was determined to be > 90% identity and similarity. The files generated by this analysis are publically available at NCBI Gene Expression Omnibus [97], accession number GSE116819.

### RT-PCR validation

RNA extraction was performed as described above. 1 μg of RNA was used to synthesize cDNA using the Qiagen quantitect reverse transcription kit (Qiagen 205,311) or the Biosystems high capacity cDNA reverse transcription kit (Thermofisher 4,368,814). RT-PCR was performed with SensiFAST SYBR No Rox (Bioline, BIO-98002) on a Bio-Rad CFX96 real time PCR system. Primer sequences are available upon request. Delta-delta CT method was used to calculate changes in expression, relative to expression levels of actin, in the PCR products.

### DAVID and ingenuity network analysis

The Human Genome Organization (HUGO) Human Gene Nomenclature Committee (HGNC) database was utilized to convert *Xenopus* probes and reads to human nomenclature (HUGO Gene Nomenclature Committee at the European Bioinformatics Institute, genenames.org). The converted *Xenopus* gene names were used for all analyses and used within the text to maintain consistency and readability.

The significantly altered genes from each dataset were imported in DAVID Bioinformatics Resources [23, 104]. Genes that were significantly altered were assessed for their molecular function using the Kyoto Encyclopedia of Genes and Genomes (KEGG) database resources [105] and Gene Ontology (GO) Molecular Function from the Gene Ontology Consortium [106]. These functions were compiled and sorted according to genetic alteration (increase vs. decrease, relative to control). To further examine the functions of these genes, they were imported into Ingenuity Pathway Analysis software and the resultant networks examined (IPA version 31813283, Qiagen, Hilden, Germany). Custom pathways were built using the My Pathway tool.

In the microarray analysis, 415 unique genes with human orthologs were altered by 1.75 fold or greater with treatment 1 RAR inhibition. In the RNAseq Analysis, 93 unique genes with human orthologs were altered significantly with RAR inhibition during the late treatment window. For each network generated in IPA, the maximum number of altered genes included in the network was set at 30 genes. This number was chosen to ensure interpretability of the network. Occasionally genes that were altered by less than 1.75 fold in treatment 1 were included in the network if their function was integral to that network.

### OMIM analysis

A keyword search was performed on the Online Mendelian Inheritance of Man (OMIM.org) to identify genes that were related to a craniofacial defect. Our search was limited to the keywords shown in Fig. 7. Three hundred and twenty five genes were identified, associated with 307 unique disorders (see Additional file 4: Table S2). These genes were compared to the genes that were significantly altered with early RAR inhibition (415 genes) and late RAR inhibition (93 genes) to identify the human disorders associated with genes altered with RAR inhibition.

## Additional files

Additional file 1: **Figure S1.** (a) Face dissection area for RAR inhibition analyses. Dissection area for early analysis is denoted with blue dots, dissection area for late analysis denoted with green dots. (b) Landmark Diagram for morphometric analyses. (TIF 427 kb)

Additional file 2: **Figure S2.** Quantitative RT-PCR validation of select genes. (TIF 208 kb)

Additional file 3 **Table S1.** Human disorders with a median craniofacial component and associated genes. Disorders that were identified by a keyword search in OMIM.org for six keywords listed in Fig. 7. The associated gene(s) are listed with their corresponding OMIM number. Multiple genes were identified for some disorders. (PDF 213 kb)

Additional file 4: **Table S2.** Genes altered with RAR inhibition. a. Decreased (blue) and increased (orange) genes after early RAR inhibition. Fold change altered by more than 1.75 fold *p* < 0.01. b. Decreased (green) and increased (purple) genes after late RAR inhibition. Log_2_ Fold Change significant *p* < 0.05. (PDF 182 kb)

## Abbreviations

ALX1: ALX homeobox 1
ALX4: ALX homeobox 4
ATP: Adenosine tri phosphate
ATRX: Alpha thalassemia/mental retardation syndrome X-linked
BGN: Biglycan
BM7: Bone morphogenetic protein 7
BMP: Bone morphogenetic protein
BMP2: Bone morphogenetic protein 2
BMP4: Bone morphogenetic protein 4
BMP5: Bone morphogenetic protein 5
BRD4: Bromodomain containing 4
CAMKIIg: Calcium/calmodulin dependent kinase 2 gamma
CHD1: Chromodomain helicase DNA binding protein 1
CHD2: Chromodomain helicase DNA binding protein 2
COL9A: Collagen, type IX alpha 1 chain
CVA: Canonical variate analysis
DFA: Discriminant function analysis
DLX2: Distal-less homeobox 2
DMSO: Dimethyl sulfoxide
DNA: Deoxyribonucleic acid
DYRK1A: Dual specificity tyrosine phosphorylation regulated kinase 1A
EZH2: Enhancer of zeste 2 polycomb repressive complex 2 subunit
FOXC1: Forkhead box C1
FOXD1: Forkhead box D1
FOXP4: Forkhead box P4
FRZB: Frizzled related protein
GATA2: GATA binding protein 2
GO: Gene Ontology
HDAC1: Histone deacetylase 1
HGNC: HUGO Gene Nomenclature Committee
HOXA4: Homeobox A4
HPF: Hours post fertilization
HUGO: Human Genome Organization
IACUC: Institutional animal care and use committee
IPA: Ingenuity Pathway Analysis
ITPR: Inositol 1,4,5-triphosphate receptors
KEGG: Kyoto Encyclopedia of Genes and Genomes
LHX1: LIM homeobox 1
MBS: Modified Barth’s saline
MTA2: Metastasis associated 1 family member 2
NCBI: National Center for Biotechnology Information
NCOA: Nuclear receptor coactivator 1
NCOA3: Nuclear receptor coactivator 3
NCOR1: Nuclear receptor corepressor 1
NFAT: Nuclear factor of activated T-cells
NFATC3: Nuclear factor of activated T cells 3
OMIM: Online Mendelian inheritance of man
PBT: Phosphate buffered saline + Tween
PCR: Polymerase chain reaction
PD: Procrustes’ distance
PFA: Paraformaldehyde
PP3CA: Protein phosphatase 3 catalytic subunit alpha
PRC2: Polycomb repressive complex 2
RA: Retinoic acid
RAR: Retinoic acid receptor
RARϒ: Retinoic acid receptor gamma
RNA: Ribonucleic acid
RNAseq: Ribonucleic acid-sequencing
RT-PCR: Real time-polymerase chain reaction
RXR: Retinoid x receptor
SIX2: SIX homeobox 2
SMARCAD1: SWI/SNF-related matrix-associated actin dependent regulator of chromatin, subfamily a, containing DEAD/H box 1
SUZ12: Suppressor of zeste 12 polycomb repressive complex 2 subunit
TGFB1: Transforming growth factor beta 1
TGFbeta: Transforming growth factor beta
VCU: Virginia Commonwealth University
WNT1: Wnt family member 1
