# Supplementary figures and images for: Transcriptome analysis of Xenopus orofacial tissues deficient in retinoic acid receptor function
Source: BMC Genomics. 2018 Nov 3;19:795. doi: 10.1186/s12864-018-5186-8 (PMC6215681; doi:10.1186/s12864-018-5186-8)

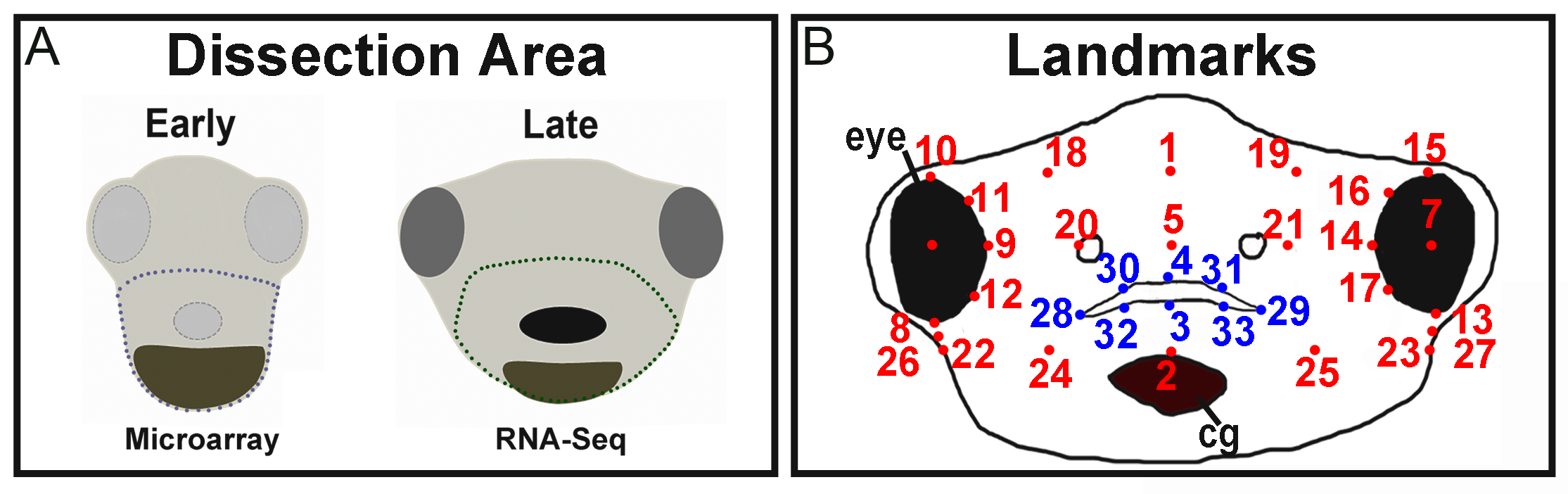

Supplement: Supplementary file 1 — Figure S1. (a) Face dissection area for RAR inhibition analyses. Dissection area for early analysis is denoted with blue dots, dissection area for late analysis denoted with green dots. (b) Landmark Diagram for morphometric analyses. (TIF 427 kb) [file 12864_2018_5186_MOESM1_ESM.tif]

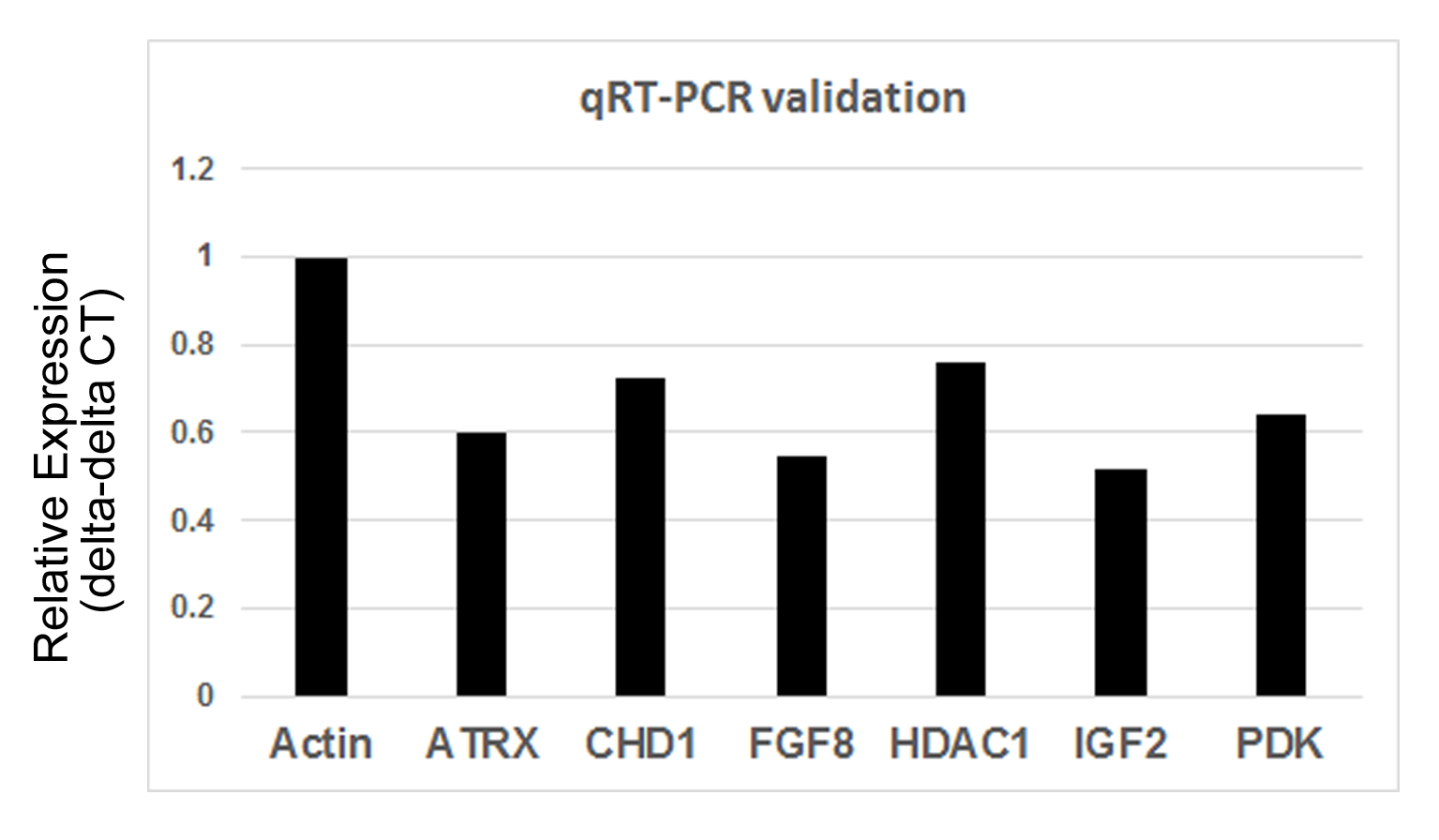

Supplement: Supplementary file 2 — Figure S2. Quantitative RT-PCR validation of select genes. (TIF 208 kb) [file 12864_2018_5186_MOESM2_ESM.tif]
