# Supplementary material for: Transcriptome analysis of Xenopus orofacial tissues deficient in retinoic acid receptor function
Source: BMC Genomics. 2018 Nov 3;19:795. doi: 10.1186/s12864-018-5186-8 (PMC6215681; doi:10.1186/s12864-018-5186-8)
Supplement: Supplementary file 3 — Table S1. Human disorders with a median craniofacial component and associated genes. Disorders that were identified by a keyword search in OMIM.org for six keywords listed in Fig. 7. The associated gene(s) are listed with their corresponding OMIM number. Multiple genes were identified for some disorders. (PDF 213 kb) [file 12864_2018_5186_MOESM3_ESM.pdf]

Table S1. Human disorders with a median craniofacial component and associated genes.
Disorders that were identified by a keyword search in OMIM.org for six keywords listed in Figure 7. The associated gene(s) are listed with their corresponding OMIM number. Multiple genes were identified for some disorders.

**Disorders Associated with OMIM Keyword Search for Craniofacial Defects**			
**Disease OMIM Number**	**Disease Name**	***Associated Gene***	**Gene OMIM Number**
100300	ADAMS-OLIVER SYNDROME 1	*ARHGAP31*	610911
100800	ACHONDROPLASIA	*FGFR3*	134934
101400	SAETHRE-CHOTZEN SYNDROME	*TWIST1*	601622
101600	PFEIFFER SYNDROME	*FGFR1*	136350
		FGFR2	176943
105650	DIAMOND-BLACKFAN ANEMIA 1	*RPS19*	603474
106260	ANKYLOBLEPHARON-ECTODERMAL DEFECTS-CLEFT LIP/PALATE	*TP63*	603273
106600	TOOTH AGENESIS, SELECTIVE, 1	*MSX1*	142983
108300	STICKLER SYNDROME, TYPE 1	*COL2A1*	120140
108720	ATELOSTEOGENESIS, TYPE 1	*FLNB*	603381
109400	BASAL CELL NEVUS SYNDROME	*PTCH1*	601309
		PTCH2	603673
		SUFU	607035
113620	BRANCHIOOCULOFACIAL SYNDROME	*TFAP2A*	107580
114290	CAMPOMELIC DYSPLASIA	*SOX9*	608160
114300	ARTHROGRYPOSIS, DISTAL, TYPE 3	*PIEZO2*	613629
117000	CENTRAL CORE DISEASE OF MUSCLE	*RYR1*	180901
117650	CEREBROCOSTOMANDIBULAR SYNDROME	*SNRPB*	182282
119300	VAN DER WOUDE SYNDROME 1	*IRF6*	607199
119500	POPLITEAL PTERYGIUM SYNDROME	*IRF6*	607199
119540	CLEFT PALATE, ISOLATED	*UBB*	191339
119600	CLEIDOCRANIAL DYSPLASIA	*RUNX2*	600211
120433	COLOBOMA, OCULAR, WITH OR WITHOUT HEARING IMPAIRMENT, CLEFT LIP/PALATE, AND/OR MENTAL RETARDATION	*YAP1*	606608
122470	CORNELIA DE LANGE SYNDROME 1	*NIPBL*	608667
123150	JACKSON-WEISS SYNDROME	*FGFR1*	136350
		FGFR2	176943
123500	CROUZAN SYNDROME	*FGFR2*	176943
124500	VOHWINKEL SYNDROME	*GJB2*	121011
129400	RAPP-HODGKIN SYNDROME	*TP63*	603273
130070	EHLERS-DANLOS SYNDROME WITH SHORT STATURE AND LIMB ANOMALIES	*B4GALT7*	604327
130650	BECKWITH-WIEDEMANN SYNDROME	*CDKN1C*	600856
		ICR1	616186
		H19	103280

		KCNQ10T1	604115
135900	COFFIN-SIRIS SYNDROME 1	*ARID1B*	614556
136760	FRONTONASAL DYSPLASIA 1	*ALX3*	606014
		KIF3A	604683
137215	GASTRIC CANCER, FAMILIAL DIFFUSE, WITH OR WITHOUT CLEFT LIP AND/OR PALATE	*CDH1*	192090
139210	MYHRE SYNDROME	*SMAD4*	600993
142945	HOLOPROSENCEPHALY 3	*SHH*	600725
142946	HOLOPROSENCEPHALY 4	*TGIF1*	602630
145410	OPITZ GBBB SYNDROME, TYPE 2	*SPECC1L*	614140
146110	HYPOGONADOTROPIC HYPOGONADISM 7 WITH OR WITHOUT ANOSMIA	*GNRHR*	138850
146255	HYPOPARATHYROIDISM, SENSORINEURAL DEAFNESS, AND RENAL DISEASE	*GATA3*	131320
146510	PALLISTER-HALL SYNDROME	*GLI3*	165240
147920	KABUKI SYNDROME 1	*KMT2D*	602113
148820	WAARDENBURG SYNDROME, TYPE 3	*PAX3*	606597
150250	LARSEN SYNDROME	*FLNB*	603381
151050	LENZ-MAJEWSKI HYPEROSTOTIC DWARFISM	*PTDSS1*	612792
153400	LYMPHEDEMA-DISTICHIASIS SYNDROME WITH RENAL DISEASE AND DIABETES MELLITUS INCLUDED	*FOXC2*	602402
154400	ACROFACIAL DYSTOSIS 1, NAGER TYPE	*SF3B4*	605593
154500	TREACHER COLLINS SYNDROME	*TCOF1*	606847
154780	MARSHALL SYNDROME	*COL11A1*	120280
156400	METAPHYSEAL CHONDRODYSPLASIA, JANSEN TYPE	*PTHR1*	168468
156550	KNIEST DYSPLASIA	*COL2A1*	120140
156610	SKIN CREASES, CONGENITAL SYMMETRIC CIRCUMFERENTIAL 1	*TUBB*	191130
157170	HOLOPROSENCEPHALY 2	*SIX3*	603714
161200	NAIL-PATELLA SYNDROME	*LMX1B*	602575
162100	AMYOTROPHY, HEREDITARY NEURALGIC	*SEPT9*	604061
163200	SCHIMMELPENNING-FEUERSTEIN-MIMS SYNDROME	*NRAS*	164790
		HRAS	190020
		KRAS	190070
164200	OCULODENTODIGITAL DYSPLASIA	*GJA1*	121014
168500	PARIETAL FORAMINA 1	*MSX2*	123101
170390	ANDERSEN CARDIODYSRHYTHMIC PERIODIC PARALYSIS	*KCNJ2*	600681
174300	OROFACIODIGITAL SYNDROME 5	*DDX59*	615464
180200	RETINOBLASTOMA	*RB1*	614041
182212	SHPRINTZEN-GOLDBERG CRANIOSYNOSTOSIS SYNDROME	*SKI*	164780
182290	SMITH-MAGENIS SYNDROME	*RAI*	607642
183900	SPONDYLOEPIPHYSEAL DYSPLASIA CONGENITA	*COL2A1*	120140
184250	SPONDYLOEPIMETAPHYSEAL DYSPLASIA STRUDWICK TYPE	*COL2A1*	120140
184840	OTOSPONDYLOMEGAEPIPHYSEAL DYSPLASIA, AUTOSOMAL DOMINANT	*COL11A2*	120290
188400	DIGEORGE SYNDROME	*TBX1*	602054
192430	VELOCARDIOFACIAL SYNDROME	*TBX1*	602054
193230	VITREORETINAL DEGENERATION, SNOWFLAKE TYPE	*KCN13*	602308
193500	WAARDENBURG SYNDROME, TYPE 1	*PAX3*	606597

193700	ARTHROGRYPOSIS, DISTAL, TYPE 2A	*MYH3*	160720
194080	DENYS-DRASH SYNDROME	*WT1*	607102
202650	AGNATHIA-OTOCEPHALY COMPLEX	*PRRX1*	167420
206900	MICROPHTHALMIA, SYNDROMIC 3	*SOX2*	184429
206920	MICROPHTHALMIA WITH LIMB ANOMALIES	*SMOC1*	608488
208150	FETAL AKINESIA DEFORMATION SEQUENCE	*RAPSN*	601592
		DOK7	610285
		MUSK	601296
209885	BARBER-SAY SYNDROME	*TWIST2*	607556
211750	C SYNDROME	*CD96*	606037
212780	CENANI-LENZ SYNDACTYLY SYNDROME	*LRP4*	604270
213980	CRANIOFACIAL DYSMORPHISM, SKELETAL ANOMALIES, AND MENTAL RETARDATION SYNDROME	*TMCO1*	614123
214300	KLIPPEL-FEIL SYNDROME 2, AUTOSOMAL RECESSIVE	*MEOX1*	600147
214800	CHARGE SYNDROME	*SEMA3E*	608166
		CHD7	608892
215045	CHONDRODYSPLASIA, BLOMSTRAND TYPE	*PTH1R*	168468
215150	OTOSPONDYLOMEGAEPIPHYSEAL DYSPLASIA, AUTOSOMAL RECESSIVE	*COL11A2*	120290
216360	COACH SYNDROME	*TMEM67*	609884
		CC2D2A	612013
		RPGRIP1L	610937
217095	CONOTRUNCAL ANOMALY FACE SYNDROME	*TBX1*	602054
219000	FRASER SYNDROME 1	*FRAS1*	607830
220111	LEIGH SYNDROME, FRENCH CANANDIAN TYPE	*LRPPRC*	607544
220210	RITSCHER-SCHINZEL SYNDROME	*WSHC5*	610657
222600	DIASTROPHIC DYSPLASIA, BROAD BONE-PLATYSPONDYLIC VARIANT INCLUDED	*SLC26A2*	606718
225060	CLEFT LIP/PALATE-ECTODERMAL DYSPLASIA SYNDROME	*NECTIN1*	600644
225300	SPLIT-HAND/FOOT MALFORMATION 6	*WNT10B*	601906
225500	ELLIS-VAN CREVELD SYNDROME	*EVC*	604831
225300	SPLIT-HAND/FOOT MALFORMATION 6	*EVC2*	607261
225790<br>227170	PROLIERATIVE VASCULOPATHY AND HYDRANENCEPHALY-HYDROCEPHALY SYNDROME<br>OROFACIAL DIGITAL SYNDROME 6	*FLVCR2*	610865
		C5ORF42	614571
228930	FIBULAR APLASIA OR HYPOPLASIA, FEMORAL BOWING AND POLY-, SYN-, OLIGODACTYLY	*WNT7A*	601570
230740	GAPO SYNDROME	*ANTXR1*	606410
235730	MOWAT-WILSON SYNDROME	*ZEB2*	605802
236100	HOLOPROSENCEPHALY 1	*GAS1*	139185
236250	HOMOCYSTINURIA DUE TO DEFICIENCY OF N(5,10)-METHYLENETERAHYDROFOLATE REDUCTASE ACTIVITY	*MTHFR*	607093
236670	MUSCULAR DYSTROPHY-DYSTROGLYCANOPATHY (CONGENITAL WITH BRAIN AND EYE ANOMALIES), TYPE A1	*POMT1*	607423
236680	HYDROLETHALUS SYNDROME 1	*HYLS1*	610693
239300	HYPERPHOSPHATASIA WITH MENTAL RETARDATION SYNDROME 1	*PIGV*	610274
241800	HYPOTHALAMIC HAMARTOMAS	*GLI3*	165240
241850	HYPOTHYROIDISM, THYROIDAL OR ATHYROIDAL, WITH SPIKY	*FOXE1*	602617

	HAIR AND CLEFT PALATE		
242840	VICI SYNDROME	*EPG5*	615086
243310	BARAITSER-WINTER SYNDROME 1	*ACTB*	102630
243605	STROMME SYNDROME	*CENPF*	600236
244200	HYPOGONADOTROPIC HYPOGONADISM 3 WITH OR WITHOUT ANOSMIA	*PROKR2*	607123
245150	KEUTEL SYNDROME	*MGP*	154870
245600	MULTIPLE JOINT DISLOCATIONS, SHORT STATURE, AND CRANIOFACIAL DYSMORPHISM WITH OR WITHOUT CONGENITAL HEART DEFECTS	*B3GAT3*	606374
248390	TREACHER COLLINS SYNDROME 3	*POLR1C*	610060
248500	MANNISOIDOSIS, ALPHA B, LYSOSOMAL	*MAN2B1*	609458
248700	MARDEN-WALKER SYNDROME	*PIEZO2*	613629
249000	MECKEL SYNDROME	*MKS1*	609883
253250	MULIBREY NANISM	*TRIM37*	605073
255320	MINICORE MYOPATHY WITH EXTERNAL OPHTHALMOPLEGIA	*RYR1*	180901
255995	NATIVE AMERICAN MYOPATHY	*STAC3*	615521
256030	NEMALINE MYOPATHY 2	*NEB*	161650
256050	ATELOSTEOGENESIS, TYPE 2	*SLC26A2*	606718
256520	NEU-LAXOVA SYNDROME 1	*PHGDH*	606879
257300	MOSAIC VARIEGATED ANEUPLOIDY SYNDROME 1	*BUB1B*	602860
257920	3MC SYNDROME 1	*MASP1*	600521
258860	OROFACIAL DIGITAL SYNDROM 4	*TCTN3*	613847
259100	HYPERTROPHIC OSTEOARTHROPATHY, PRIMARY, AUTOSOMAL RECESSIVE 1	*HPGD*	601688
259775	RAINE SYNDROME	*FAM20C*	611061
260660	COUSIN SYNDROME	*TBX15*	604127
261540	PETERS-PLUS SYNDROME	*B3GLCT*	610308
262500	LARON SYNDROME	*GHR*	600946
263520	SHORT-RIB THORACIC DSYPLASIA 6 WITH OR WITHOUT POLYDACTYLY	*NEK1*	604588
263650	POPLITEAL PTERYGIUM SYNDROME, LETHAL TYPE	*RIPK4*	605706
263750	POSTAXIAL ACROFACIAL DYSOSTOSIS	*DHODH*	126064
265000	MULTIPLE PTERYGIUM SYNDROME, ESCOBAR VARIANT	*CHRNG*	100730
265050	3MC SYNDROME 2	*COLEC11*	612502
266280	RAPADILINO SYNDROME	*RECQL4*	603780
266920	SHORT-RIB THORACIC DYSPLASIA 9 WITH OR WITHOUT POLYDACTYLY	*IFT140*	614620
267000	PERLMAN SYNDROME	*DIS3L2*	614184
268300	ROBERTS SYNDROME	*ESCO2*	609353
268310	ROBINOW SYNDROME, AUTOSOMAL RECESSIVE	*ROR2*	602337
268305	ROBIN SEQUENCE WITH CLEFT MANDIBLE AND LIMB ANOMALIES	*EIF4A3*	608546
268400	ROTHMUND-THOMSON SYNDROME	*RECQL4*	603780
269000	SC PHOCOMELIA SYNDROME	*ESCO2*	609353
270400	SMITH-LEMLI-OPTIZ SYNDROME	*DCHR7*	602858
270420	DIARRHEA 3, SECRETORY SODIUM, CONGENITAL, WITH OR WITHOUT OTHER CONGENITAL ANOMALIES	*SPINT2*	605124

271640	SPONDYLOEPIMETAPHYSEAL DYSPLASIA WITH JOINT LAXITY, TYPE 1, WITH OR WITHOUT FRACTURES	*B3GALT6*	615291
272430	COLD-INDUCED SWEATING SYNDROME	*CRLF1*	604237
272440	FILIPPI SYNDROME	*CKAP2L*	616174
272460	SPONDYLCARPOTARSAL SYNOSTOSIS SYNDROME	*FLNB*	603381
273395	TETRAAMELIA SYNDROME, AUTOSOMAL RECESSIVE; TETAMS	*WNT3*	165330
276820	ULNA AND FIBULA, ABSENCE OF, WITH SEVERE LIMB DEFICIENCY	*WNT7A*	601570
276950	VACTERL ASSOCIATION WITH HYDROCEPHALUS	*PTEN*	601728
277450	VITAMIN K-DEPENDENT CLOTTING FACTORS, COMBINED DEFICIENCY OF, 1	*GGCX*	137167
280000	COLOBOMA, CONGENITAL HEART DISEASE, ICHTHYOSIFORM DERMATOSIS, MENTAL RETARDATION, AND EAR ANOMALIES SYNDROME	*PIGL*	605947
300000	OPITZ GBBB SYNDROME, TYPE I	*MID1*	300552
300004	CORPUS CALLOSUM, AGENESIS OF, WITH ABNORMAL GENITALIA	*ARX*	300382
300166	MICROPHTHALMIA, SYNDROMIC 2	*BCOR*	300485
300209	SIMPSON-GOLABI-BEHMEL SYNDROME, TYPE 2	*OFD1*	300170
300215	LISSENCEPHALY, X-LINKED, 2	*ARX*	300382
300244	TERMINAL OSSEOUS DYSPLASIA	*FLNA*	300017
300263	SIDERIUS X-LINKED MENTAL RETARDATION SYNDROME	*PHF8*	300560
300321	FG SYNDROME 2	*FLNA*	300017
300373	OSTEOPATHIA STRIATA WITH CRANIAL SCLEROSIS	*AMER1*	300647
300590	CORNELIA DE LANGE SYNDROME 2	*SMC1A*	300040
300749	MENTAL RETARDATION AND MICROCEPHALY WITH PONTINE AND CEREBELLAR HYPOPLASIA	*CASK*	300172
300804	JOUBERT SYNDROME 10	*OFD1*	300170
300867	KABUKI SYNDROME 2	*KMD6A*	300128
300868	MULTIPLE CONGENITAL ANOMALIES-HYPOTONIA-SEIZURES SYNDROME 2	*PIGA*	311770
300882	CORNELIA DE LANGE SYNDROME 5	*HDAC8*	300269
300946	DIAMOND-BLACKFAN ANEMIA 14 WITH MANDIBULOFACIAL DYSOSTOSIS	*TSR2*	300945
300958	MENTAL RETARDATION, X-LINKED 102	*DDDX3X*	300160
300960	MEND SYNDROME	*EBP*	300205
302905	ABRUZZO-ERICKSON SYNDROME	*TBX22*	300307
303400	CLEFT PALATE WITH OR WITHOUT ANKYLOGLOSSIA, X-LINKED	*TBX22*	300307
304110	CRANIOFRONTONASAL SYNDROME	*EFNB1*	300035
304120	OTOPALATODIGITAL SYNDROME, TYPE II	*FLNA*	300017
305600	FOCAL DERMAL HYPOPLASIA	*PORCN*	300651
305620	FRONTOMETAPHYSEAL DYSPLASIA 1	*FLNA*	300017
307000	HYDROCEPHALUS DUE TO CONGENITAL STENOSIS OF AQUEDUCT OF SYLVIUS	*L1CAM*	308840
308205	IFAP SYNDROME WITH OR WITHOUT BRESHECK SYNDROME	*MBTPS2*	300294
308700	HYPOGONADOTROPIC HYPOGONADISM 1 WITH OR WITHOUT ANOSMIA	*ANOS1*	300836
309350	MELNICK-NEEDLES SYNDROME	*FLNA*	300017
309500	RENPENNING SYNDROME 1	*PQBP1*	300463

309520	LUJAN-FRYNS SYNDROME	*MED12*	300188
309580	MENTAL RETARDATION-HYPOTONIC FACIES SYNDROME, X-LINKED, 1	*ATRX*	300032
309583	MENTAL RETARDATION, X-LINKED, SYNDROMIC, SNYDER-ROBINSON TYPE	*SMS*	300105
311200	OROFACIODIGITAL SYNDROME I; OFD1	*OFD1*	300170
311300	OTOPALATODIGITAL SYNDROME, TYPE I	*FLNA*	300017
311900	TARP SYNDROME	*RBM10*	300080
312870	SIMPSON-GOLABI-BEHMEL SYNDROME, TYPE 1	*GPC3*	300037
314390	VACTERL ASSOCIATION, X-LINKED, WITH OR WITHOUT HYDROCEPHALUS	*ZIC3*	300265
400044	46,XY SEX REVERSAL 1	*SRY*	480000
600251	FACIAL CLEFTING, OBLIQUE, 1	*SPECC1L*	614140
600625	OROFACIAL CLEFT 11	*BMP4*	112262
600920	VAN DEN ENDE-GUPTA SYNDROME	*SCARF2*	613619
601186	MICROPHTHALMIA, SYNDROMIC 9	*STRA6*	610745
601492	MUCOPOLYSACCHARIDOSIS, TYPE 9	*HYAL1*	607071
601680	ARTHROGRYPOSIS, DISTAL	*MYH3*	160720
		TPM2	190990
		TNNI2	191043
		TNNT3	600692
601776	EHLERS-DANLOS SYNDROME, MUSCULOCONTRACTURAL TYPE 1	*CHST14*	608429
602081	SPEECH-LANGUAGE DISORDER 1	*FOXP2*	605317
602361	GRACILE BONE DYSPLASIA	*FAM111A*	615292
602483	AURICULOCONDYLAR SYNDROME 1	*GNAI3*	139370
602849	MUENKE SYNDROME	*FGFR3*	134934
603194	MECKEL SYNDROME, TYPE 2	*TMEM216*	613277
603543	LIMB-MAMMARY SYNDROME	*TP63*	603273
603546	SPONDYLOEPIMETAPHYSEAL DYSPLASIA WITH JOINT LAXITY, TYPE 2	*KIF22*	603213
603671	ACROMELIC FRONTONASAL DYSOSTOSIS	*ZSWIM6*	615951
604232	LEBER CONGENITAL AMAUROSIS 3	*SPATA7*	609868
604292	ECTRODACTYLY, ECTODERMAL DYSPLASIA, AND CLEFT LIP/PALATE SYNDROME 3	*TP63*	603273
604841	STICKLER SYNDROME, TYPE II	*COL11A1*	120280
605039	BOHRING-OPITZ SYNDROME	*ASXL1*	612990
605282	TEMTAMY PREAXIAL BRACHYDACTYLY SYNDROME	*CHSY1*	608183
605289	SPLIT-HAND/FOOT MALFORMATION 4	*TP63*	603273
606164	DIAMOND-BLACKFAN ANEMIA 15 WITH MANDIBULOFACIAL DYSOSTOSIS	*RPS28*	603685
606170	GENITOPATELLAR SYNDROME	*KAT6B*	605880
606713	VAN DER WOUDE SYNDROME 2	*GRHL3*	608317
607313	GAZE PALSY, FAMILIAL HORIZONTAL, WITH PROGRESSIVE SCOLIOSIS, 1	*ROBO3*	608630
607330	LATHOSTEROLOSIS	*SC5D*	602286
607371	DYSTONIA, JUVENILE-ONSET	*ACTB*	102630
607812	CRANIOLENTICULOSUTURAL DYSPLASIA	*SEC23A*	610511
607932	MICROPHTHALMIA, SYNDROMIC 6	*BMP4*	112262

608572	BURN-MCKEOWN SYNDROME	*TXNL4A*	611595
608864	OROFACIAL CLEFT 6, SUSCEPTIBILITY TO	*IRF6*	607199
608874	OROFACIAL CLEFT 5	*MSX1*	142983
609192	LOEYS-DIETZ SYNDROME 1	*TGFBR1*	190181
609460	GOLDBERG-SHPRINTZEN SYNDROME	*KIAA1279*	609367
609597	PARIETAL FORAMINA 2	*ALX4*	605420
609637	HOLOPROSENCEPHALY 5	*ZIC2*	603073
610125	MICROPHTHALMIA, SYNDROMIC 5	*OTX2*	600037
610168	LOEYS-DIETZ SYNDROME 2	*TGFBR2*	190182
610443	KOOLEN-DE VRIES SYNDROME	*KANSL1*	612452
610536	MANDIBULOFACIAL DYSOSTOSIS, GUION-ALMEIDA TYPE	*EFTUD2*	603892
610628	HYPOGONADOTROPIC HYPOGONADISM 4 WITH OR WITHOUT ANOSMIA	*PROK2*	607002
610759	CORNELIA DE LANGE SYNDROME 3	*SMC3*	606062
610828	HOLOPROSENCEPHALY 7	*PTCH1*	601309
610829	HOLOPROSENCEPHALY 9	*GLI2*	165230
611263	SHORT-RIB THORACIC DYSPLASIA 2 WITH OR WITHOUT POLYDACTYLY	*IFT80*	611177
611561	MECKEL SYNDROME, TYPE 5	*RPGRIP1L*	610397
611881	GLYCOGEN STORAGE DISEASE, XII	*ALDOA*	103850
612164	EPILEPTIC ENCEPHALOPATHY, EARLY INFANTILE, 4; EIEE4	*STXBP1*	602926
612284	MECKEL SYNDROM, TYPE 6	*CC2D2A*	612013
612290	MICROTIA, HEARING IMPAIRMENT, AND CLEFT PALATE	*HOXA2*	604685
612313	GLASS SYNDROME	*SATB2*	608148
612370	HYPOGONADOTROPIC HYPOGONADISM 5 WITH OR WITHOUT ANOSMIA	*CHD7*	608892
612527	DIAMOND-BLACKFAN ANEMIA 4	*RPS17*	180472
612541	NEUTROPENIA, SEVERE CONGENITAL 4, AUTOSOMAL RECESSIVE	*G6PC3*	611045
612561	DIAMOND-BLACKFAN ANEMIA 6	*RPL5*	603634
612562	DIAMOND-BLACKFAN ANEMIA 7	*RPL11*	604175
612563	DIAMOND-BLACKFAN ANEMIA 8	*RPS7*	603658
612651	ENDOCRINE-CEREBROOSTEODYSPLASIA	*ICK*	612325
612702	HYPOGONADOTROPIC HYPOGONADISM 6 WITH OR WITHOUT ANOSMIA	*FGF8*	600483
613038	PITUITARY HORMONE DEFICIENCY, COMBINED, 1	*POU1F1*	173110
613091	SHORT-RIB THORACIC DYSPLASIA 3 WITH OR WITHOUT POLYDACTYLY	*DYNC2H1*	603297
613150	MUSCULAR DYSTROPHY-DYSTROGLYCANOPATHY (CONGENITAL WITH BRAIN AND EYE ANOMALIES), TYPE A, 2	*POMT2*	607439
613192	MENTAL RETARDATION, AUTOSOMAL RECESSIVE 13	*TRAPPC9*	611966
613309	DIAMOND-BLACKFAN ANEMIA 10	*RPS26*	603701
613451	FRONTONASAL DYSPLASIA 2	*ALX4*	605420
613456	FRONTONASAL DYSPLASIA 3	*ALX1*	601527
613705	OROFACIAL CLEFT 10	*SUMO1*	601912
613717	TREACHER COLLINS SYNDROME 2	*POLR1D*	613715
613805	MEIER-GORLIN SYNDROME 5	*CDC6*	602627
613819	SHORT-RIB THORACIC DYSPLASIA 4 WITH OR WITHOUT POLYDACTYLY	*TTC21B*	612014

613885	MECKEL SYNDROME, TYPE 8	*TCTN2*	613846
614069	IMMUNODEFICIENCY-CENTROMERIC INSTABILITY-FACIAL ANOMALIES SYNDROME 2	*ZBTB24*	614064
614078	CHONDRODYSPLASIA WITH JOINT DISLOCATIONS, GPAPP TYPE	*IMPAD1*	614010
614091	SHORT-RIB THORACIC DYSPLASIA 7 WITH OR WITHOUT POLYDACTYLY	*WDR35*	613602
614120	HYDROLETHALUS SYNDROME 2	*KIF7*	611254
614207	HYPERPHOSPHATASIA WITH MENTAL RETARDATION SYNDROME 3	*PGAP2*	615187
614226	HOLOPROSENCEPHALY 11	*CDON*	608707
614261	MICROCEPHALY-CAPILLARY MALFORMATION SYNDROME; MICCAP	*STAMPBP*	606247
614376	SHORT-RIB THORACIC DYSPLASIA 5 WITH OR WITHOUT POLYDACTYLY	*WDR19*	608151
614399	MYOPATHY, AREFLEXIA, RESPIRATORY DISTRESS, AND DYSPHAGIA, EARLY-ONSET	*MEGF10*	612453
614402	MICROPHTHALMIA, SYNDROMIC 11	*VAX1*	604294
614583	BARAITSER-WINTER SYNDROME 2	*ACTG1*	102560
614669	AURICULOCONDYLAR SYNDROME 2	*PLCB4*	600810
614732	INTRAUTERINE GROWTH RETARDATION, METAPHYSEAL DYSPLASIA, ADRENAL HYPOPLASIA CONGENITA, AND GENITAL ANOMALIES	*CDKN1C*	600856
614838	HYPOGONADOTROPIC HYPOGONADISM 9 WITH OR WITHOUT ANOSMIA	*NSMF*	608137
614880	HYPOGONADOTROPIC HYPOGONADISM 15 WITH OR WITHOUT ANOSMIA	*HS6ST1*	605846
614900	DIAMOND-BLACKFAN ANEMIA 11	*RPL26*	603704
614921	CONGENITAL DISORDER OF GLYCOSYLATION, TYPE It	*PGM1*	171900
614924	COMBINED OXIDATIVE PHOSPHORYLATION DEFICIENCY 12	*EARS2*	612799
614974	FOCAL FACIAL DERMAL DYSPLASIA 4	*CYP26C1*	608428
615270	HYPOGONADOTROPIC HYPOGONADISM 20 WITH OR WITHOUT ANOSMIA	*FGF17*	603725
615271	HYPOGONADOTROPIC HYPOGONADISM 21 WITH OR WITHOUT ANOSMIA	*FLRT3*	604808
615502	MENTAL RETARDATION, AUTOSOMAL DOMINANT 21	*CTCF*	604167
615503	SHORT-RIB THORACIC DYSPLASIA 8 WITH OR WITHOUT POLYDACTYLY	*WDR60*	516462
615524	MICROPHTHALMIA, SYNDROMIC 12	*RARB*	180220
615582	LOEYS-DIETZ SYNDROME 5	*TGFB3*	190230
615630	SHORT-RIB THORACIC DYSPLASIA 10 WITH OR WITHOUT POLYDACTYLY	*IFT172*	607386
615633	SHORT-RIB THORACIC DYSPLASIA 11 WITH OR WITHOUT POLYDACTYLY	*WDR34*	613363
615824	MITOCHONDRIAL COMPLEX III DEFICIENCY, NUCLEAR TYPE 7	*RPS7*	603658
615948	OROFACIODIGITAL SYNDROME XIV	*C2CD3*	615944
616030	HYPOGONADOTROPIC HYPOGONADISM 22 WITH OR WITHOUT ANOSMIA	*FEZF1*	613301
616038	NEU-LAXOVA SYNDROME 2	*PSAT1*	610936
616145	CATEL-MANZKE SYNDROME	*TGDS*	616146
616268	MENTAL RETARDATION, AUTOSOMAL DOMINANT 32	*KAT6A*	601408
616331	ROBINOW SYNDROME, AUTOSOMAL DOMINANT 2	*DVL1*	601365

616367	MANDIBULOFACIAL DYSTOSIS WITH ALOPECIA	*EDNRA*	131243
616449	BASEL-VANAGAITE-SMIRIN-YOSEF SYNDROME	*MED25*	610197
616462	ACROFACIAL DYSOSTOSIS, CINCINNATI TYPE	*POLR1A*	616404
616546	SHORT-RIB THORACIC DYSPLASIA 14 WITH POLYDACTYLY	*KIAA0196*	610657
616570	CEREBROOCULOFACIOSKELETAL SYNDROME 3	*ERCC5*	1333530
616728	CLEFT PALATE, PSYCHOMOTOR RETARDATION, AND DISTINCTIVE FACIAL FEATURES	*KDM1A*	609132
616734	SKIN CREASES, CONGENITAL SYMMETRIC CIRCUMFERENTIAL 2	*MAPRE2*	605789
616784	JOUBERT SYNDROME 26	*KIAA0556*	616650
616788	OROFACIAL CLEFT 15	*DLX4*	601911
616789	MENTAL RETARDATION AND DISTINCTIVE FACIAL FEATURES WITH OR WITHOUT CARDIAC DEFECTS	*MED13L*	608771
616835	MEIER-GORLIN SYNDROME 6	*GMNN*	602842
616920	HEART AND BRAIN MALFORMATION SYNDROME	*SMG9*	613176
616897	OSTEOCHONDRODYSPLASIA, COMPLEX LETHAL, SYMOENS-BARNES-GISTELINCK TYPE	*TAPT1*	612758
617667	FRASER SYNDROME 3	*GRIP1*	604597
