# Supplementary material for: Transcriptome analysis of Xenopus orofacial tissues deficient in retinoic acid receptor function
Source: BMC Genomics. 2018 Nov 3;19:795. doi: 10.1186/s12864-018-5186-8 (PMC6215681; doi:10.1186/s12864-018-5186-8)
Supplement: Supplementary file 4 — Table S2. Genes altered with RAR inhibition. a. Decreased (blue) and increased (orange) genes after early RAR inhibition. Fold change altered by more than 1.75 fold p < 0.01. b. Decreased (green) and increased (purple) genes after late RAR inhibition. Log2 Fold Change significant p < 0.05. (PDF 182 kb) [file 12864_2018_5186_MOESM4_ESM.pdf]

A.

Gene	Full Name	Fold Change
	DECREASED	
ABCC5	ATP Binding Cassette Subfamily C Member 5	-2.179
ACSBG2	Acyl-Coa Synthetase Bubblegum Family Member 2	-1.775
ACSL3	Acyl-Coa Synthetase Long Chain Family Member 3	-2.113
AFDN	Afadin, Adherens Junction Formation Factor	-1.852
ANKMY2	Ankyrin Repeat And MYND Domain Containing 2	-2.160
ANKRD11	Ankyrin Repeat Domain 11	-1.867
ANKRD12	Ankyrin Repeat Domain 12	-1.920
AP5M1	Adaptor Related Protein Complex 5 Mu 1 Subunit	-1.755
ATF7IP	Activating Transcription Factor 7 Interacting Protein	-2.203
ATL3	Atlastin GTPase 3	-1.789
ATRX	ATRX, Chromatin Remodeler	-1.977
ATXN7L3	Ataxin 7 Like 3	-1.792<br>-2.009<br>-2.037
BAK1	BCL2 Antagonist/Killer 1	-1.950
BARD1	BRCA1 Associated RING Domain 1	-2.029
BBX	BBX, HMG-Box Containing	-1.787<br>-2.011
CAMK2G	Calcium/Calmodulin Dependent Protein Kinase II Gamma	-1.773<br>-1.855<br>-1.891<br>-1.901<br>-2.102<br>-2.123<br>-2.174<br>-2.185<br>-2.202<br>-2.596
CAPN8	Calpain 8	-2.035
CCDC71L	Coiled-Coil Domain Containing 71 Like	-1.947
CDC42EP4	CDC42 Effector Protein 4	-1.881
CDH20	Cadherin 20	-1.951
CETN1	Centrin 1	-2.039
CHD1	Chromodomain Helicase DNA Binding Protein 1	-2.135<br>-2.210
CHD2	Chromodomain Helicase DNA Biding Protein 2	-1.899
CHFR	Checkpoint With Forkhead And Ring Finger Domains	-1.792
CIR1	Corepressor Interacting With RBPJ, 1	-2.196
CKAP2	Cytoskeleton Associated Protein 2	-2.615
CNOT6	CCR4-NOT Transcription Complex Subunit 6	-1.768
CRYBG1	Crystallin Beta-Gamma Domain Containing 1	-1.755
DAAM1	Dishevelled Associated Activator Of Morphogenesis 1	-1.779
DAZAP2	DAZ Associated Protein 2	-1.754
DDX10	DEAD-Box Helicase 10	-1.858
DHX16	DEAH-Box Helicase 16	-2.159
DNAJB2	DNAJ Heat Shock Protein Family (Hsp40) Member B2	-1.944
DNAJB9	DNAJ Heat Shock Protein Family (Hsp40) Member B9	-2.470
EDN1	Endothelin 1	-1.851
EFNB2	Ephrin B2	-2.326
EIF3A	Eukaryotic Translation Initiation Factor 3 Subunit A	-1.939

		-2.139<br>-2.293
EIF4E3	Eukaryotic Translation Initiation Factor 4E Family Member 3	-1.777
EIF5B	Eukaryotic Translation Initiation Factor 5B	-1.975
ELOA	Elongin A	-1.796
ESCO1	Establishment Of Sister Chromatid Cohesion N-Acetyltransferase 1	-1.947
ESPL1	Extra Spindle Pole Bodies Like 1, Separase	-1.818
EZH2	Enhancer Of Zeste 2 Polycomb Repressive Complex 2 Subunit	-1.771
FAF2	Fas Associated Factor Family Member 2	-1.862
GCLC	Glutamate-Cysteine Ligase Catalytic Subunit	-1.863<br>-1.931<br>-1.942<br>-2.025
GOLGA4	Golgin A4	-1.948
HDAC1	Histone Deacetylase 1	-1.788
HIC2	HIC ZBTB Transcriptional Repressor 2	-2.087
HMBOX1	Homeobox Containing 1	-1.939
HSPH1	Heat Shock Protein Family H (Hsp110) Member 1	-1.841
IGHV4-31	Immunoglobulin Heavy Variable 4-31	-1.896
ILF3	Interleukin Enhancer Binding Factor 3	-1.830
KIF21A	Kinesin Family Member 21A	-1.971
KLF6	Kruppel Like Factor 6	-1.960
KNOP1	Lysine Rich Nucleolar Protein 1	-1.801
KTN1	Kinectin 1	-2.010
LARP7	La Ribonucleoprotein Domain Family Member 7	-2.308
LETM2	Leucine Zipper And EF-Hand Containing Transmembrane Protein 2	-1.915
LHX8	LIM Homeobox 8	-2.027<br>-2.188
LIN28B	Lin-28 Homolog B	-1.902
LMNB1	Lamin B1	-1.913
LUC7L3	LUC7 Like 3 Pre-Mrna Splicing Factor	-2.610
MAP1LC3A	Microtubule Associated Protein 1 Light Chain 3 Alpha	-2.133
MCPH1	Microcephalin 1	-2.206
METAP1	Methionyl Aminopeptidase 1	-1.778
MPHOSPH8	M-Phase Phosphoprotein 8	-1.755<br>-1.786<br>-1.906<br>-1.921
MTIF3	Mitochondrial Translational Initiation Factor 3	-1.753
MYO6	Myosin VI	-1.869
NAA25	N(Alpha)-Acetyltransferase 25, Natb Auxiliary Subunit	-1.929
NCL	Nucleolin	-2.277<br>-2.625
NOP14	NOP14 Nucleolar Protein	-2.018
NRARP	NOTCH Regulated Ankyrin Repeat Protein	-2.057
NRG1	Neuregulin 1	-2.185
NSRP1	Nuclear Speckle Splicing Regulatory Protein 1	-2.176
ORAI2	ORAI Calcium Release-Activated Calcium Modulator 2	-1.882
OS9	OS9, Endoplasmic Reticulum Lectin	-1.812
OTOG	Otogelin	-2.032
PHF20	PHD Finger Protein 20	-1.877
PLA2G4A	Phospholipase A2 Group IVA	-1.899
PPIG	Peptidylprolyl Isomerase G	-1.790

PPP3CA	Protein Phosphatase 3 Catalytic Subunit Alpha	-2.035<br>-2.053
PRDM2	PR/SET Domain 2	-1.812
PRPF4B	Pre-mRNA Processing Factor 4B	-1.934
PRR12	Proline Rich 12	-2.103<br>-2.111
PSAT1	Phosphoserine Aminotransferase 1	-2.453
RAB27A	RAB27A, Member RAS Oncogene Family	-2.071
RABEP1	Rabaptin, RAB Gtpase Binding Effector Protein 1	-1.866<br>-2.323
RAD23B	RAD23 Homolog B, Nucleotide Excision Repair Protein	-1.935
RBM25	RNA Binding Motif Protein 25	-2.220
RBM28	RNA Binding Motif Protein 28	-1.781
RDX	Radixin	-1.934
REST	RE1 Silencing Transcription Factor	-1.795
RFK	Riboflavin Kinase	-1.829
RNF220	Ring Finger Protein 220	-1.929
RNF40	Ring Finger Protein 40	-1.903
RSF1	Remodeling And Spacing Factor 1	-1.838<br>-2.099<br>-1.944
RTF1	RTF1 Homolog, Paf1/RNA Polymerase II Complex Component	-2.017
SEMA4B	Semaphorin 4B	-2.087
SLC7A6OS	Solute Carrier Family 7 Member 6 Opposite Strand	-1.840
SMARCAD1	SWI/SNF-Related, Matrix-Associated Actin-Dependent Regulator Of Chromatin, Subfamily A, Containing DEAD/H Box 1	-1.854
SMUG1	Single-Strand-Selective Monofunctional Uracil-DNA Glycosylase 1	-1.949
SNX2	Sorting Nexin 2	-1.937
SP3	Sp3 Transcription Factor	-1.871
SPECC1	Sperm Antigen With Calponin Homology And Coiled-Coil Domains 1	-2.042
SPTBN2	Spectrin Beta, Non-Erythrocytic 2	-2.118
SRPK2	SRSF Protein Kinase 2	-1.895<br>-1.979
STK3	Serine/Threonine Kinase 3	-1.813
STRIP1	Striatin Interacting Protein 1	-1.797
SUN1	Sad1 And UNC84 Domain Containing 1	-1.836
SUPT6H	SPT6 Homolog, Histone Chaperone	-2.116<br>-2.147
SYNCRIP	Synaptotagmin Binding Cytoplasmic RNA Interacting Protein	-1.789
TBCCD1	TBCC Domain Containing 1	-1.769
THOC2	THO Complex 2	-1.779<br>-1.799
TRAPPC2	Trafficking Protein Particle Complex 2	-1.775
TRIM29	Tripartite Motif Containing 29	-2.354
TRIT1	tRNA Isopentenyltransferase 1	-2.084
TRMT10C	tRNA Methyltransferase 10C, Mitochondrial Rnase P Subunit	-2.079<br>-1.957
UBP1	Upstream Binding Protein 1	-1.900
UFL1	UFM1 Specific Ligase 1	-2.037
UHRF1	Ubiquitin Like With PHD And Ring Finger Domains 1	-2.005
USO1	USO1 Vesicle Transport Factor	-2.294
USP36	Ubiquitin Specific Peptidase 36	-1.913

VCAN	Versican	-1.781
XPO1	Exportin 1	-2.202
XRN2	5'-3' Exoribonuclease 2	-1.756
ZDHHC1	Zinc Finger DHHC-Type Containing 1	-1.851
ZFP106	Zinc Finger Protein 106	-1.816
ZNF292	Zinc Finger Protein 292	-1.823
ZNF462	Zinc Finger Protein 462	-1.912
ZNF516	Zinc Finger Protein 516	-1.868
INCREASED		
ACCS	1-Aminocyclopropane-1-Carboxylate Synthase Homolog	1.835
ACIN1	Apoptotic Chromatin Condensation Inducer 1	2.397<br>1.793<br>1.777
ACVR1B	Activin A Receptor Type 1b	1.867
ADAM9	ADAM Metallopeptidase Domain 9	1.764
AK1	Adenylate Kinase 1	2.412<br>1.763
ALAD	Aminolevulinate Dehydratase	2.125
ALDH3B1	Aldehyde Dehydrogenase 3 Family Member B1	1.911
ALDOA	Aldolase, Fructose-Bisphosphate A	2.009
ALDOB	Aldolase, Fructose-Bisphosphate B	2.383<br>1.836
AMOTL1	Angiomotin Like 1	1.910
ANGPTL7	Angiopoietin Like 7	5.013<br>2.043
AP1S1	Adaptor Related Protein Complex 1 Sigma 1 Subunit	1.789
APOBEC2	Apolipoprotein B mRNA Editing Enzyme Catalytic Subunit 2	2.229
APOH	Apolipoprotein H	2.544
APOM	Apolipoprotein M	1.944
ARHGEF19	Rho Guanine Nucleotide Exchange Factor 19	1.925
ARID3A	AT-Rich Interaction Domain 3A	2.411<br>1.986<br>1.800
ARIH2	Ariadne RBR E3 Ubiquitin Protein Ligase 2	1.956
ARRDC1	Arrestin Domain Containing 1	2.308
ATP6AP1	ATPase H+ Transporting Accessory Protein 1	1.796
ATP6V0D2	ATPase H+ Transporting V0 Subunit D2	1.852
ATP6V1B1	ATPase H+ Transporting V1 Subunit B1	2.086
ATP6V1G3	ATPase H+ Transporting V1 Subunit G3	1.949
ATXN2	Ataxin 2	2.280
BAG6	BCL2 Associated Athanogene 6	1.783
BCL9	B Cell CLL/Lymphoma 9	2.405<br>1.812
BHLHE40	Basic Helix-Loop-Helix Family Member E40	2.154
BICRA	BRD4 interacting chromatin remodeling complex associated protein	1.870
BIN1	Bridging Integrator 1	1.991<br>1.942
BMP2	Bone Morphogenetic Protein 2	2.232<br>2.220
BRD4	Bromodomain Containing 4	1.997
C2ORF76	Chromosome 2 Open Reading Frame 76	1.766
C7	Complement C7	1.763
C8B	Complement C8 Beta Chain	1.868
CA12	Carbonic Anhydrase 12	2.136

CACNA1S	Calcium Voltage-Gated Channel Subunit Alpha1 S	2.160
CACNB1	Calcium Voltage-Gated Channel Auxiliary Subunit Beta 1	2.293<br>1.870
CAPNS1	Calpain Small Subunit 1	2.546
CAPRIN1	Cell Cycle Associated Protein 1	1.991<br>1.814
CASQ1	Calsequestrin 1	1.846
CAV2	Caveolin 2	2.093<br>1.947
CAVIN1	Caveolae Associated Protein 1	1.921
CCNF	Cyclin F	2.216
CD44	CD44 Molecule (Indian Blood Group)	2.129
CDIP1	Cell Death Inducing P53 Target 1	1.783
CDX2	Caudal Type Homeobox 2	2.467
CEBPA	CCAAT/Enhancer Binding Protein Alpha	2.189
CEBPD	CCAAT/Enhancer Binding Protein Delta	1.918
CHRNA1	Cholinergic Receptor Nicotinic Alpha 1 Subunit	1.980
CHRND	Cholinergic Receptor Nicotinic Delta Subunit	1.785
CHRNG	Cholinergic Receptor Nicotinic Gamma Subunit	2.348
CKMT1B	Creatine Kinase, Mitochondrial 1B	2.004
CNOT4	CCR4-NOT Transcription Complex Subunit 4	2.005
COBL	Cordon-Bleu WH2 Repeat Protein	2.160
COL11A1	Collagen Type XI Alpha 1 Chain	1.956
COL1A1	Collagen Type I Alpha 1 Chain	2.206<br>1.999
COL1A2	Collagen Type I Alpha 2 Chain	1.776
COL28A1	Collagen Type XXVIII Alpha 1 Chain	1.984
COL2A1	Collagen Type II Alpha 1 Chain	2.239
COL5A1	Collagen Type V Alpha 1 Chain	2.211
COL9A1	Collagen Type IX Alpha 1 Chain	2.468<br>2.329
COL9A2	Collagen Type IX Alpha 2 Chain	1.769
COL9A3	Collagen Type IX Alpha 3 Chain	1.750
CPSF1	Cleavage And Polyadenylation Specific Factor 1	2.009
CRABP2	Cellular Retinoic Acid Binding Protein 2	1.824
CRP	C-Reactive Protein	2.050
CSNK2A1	Casein Kinase 2 Alpha 1	2.583<br>2.009<br>1.988
CTR9	CTR9 Homolog, Paf1/RNA Polymerase II Complex Component	1.769
DBN1	Drebrin 1	2.027
DBNL	Drebrin Like	1.792
DDT	D-Dopachrome Tautomerase	2.075
DGKA	Diacylglycerol Kinase Alpha	1.792
DHRS3	Dehydrogenase/Reductase 3	2.533
DNAJC5	Dnaj Heat Shock Protein Family (Hsp40) Member C5	1.945
DOK1	Docking Protein 1	2.037
EFNA1	Ephrin A1	2.266
EFNB1	Ephrin B1	1.919
EIF4E3	Eukaryotic Translation Initiation Factor 4E Family Member 3	2.149
ELOB	Elongin B	2.582<br>2.506
ENO3	Enolase 3	2.316
ENTPD2	Ectonucleoside Triphosphate Diphosphohydrolase 2	1.883
F11R	F11 Receptor	1.771

F2	Coagulation Factor II, Thrombin	1.805
FAM32A	Family With Sequence Similarity 32 Member A	1.938
FBLIM1	Filamin Binding LIM Protein 1	1.988
FBXL22	F-box and leucine rich repeat protein 22	1.766
FETUB	Fetuin B	2.179
FGA	Fibrinogen Alpha Chain	2.245
FLNC	Filamin C	1.969
FOS	Fos Proto-Oncogene, AP-1 Transcription Factor Subunit	1.789
FOXD1	Forkhead Box D1	2.488
FSTL1	Follistatin Like 1	1.864
G6PC	Glucose 6-Phosphatases, Catalytic	2.296
GAMT	Guanidinoacetate N-Methyltransferase	2.055<br>1.987
GATA2	GATA Binding Protein 2	2.278<br>1.846
GATA5	GATA Binding Protein 5	2.044
GEMIN2	Gem Nuclear Organelle Associated Protein 2	1.805
GJA3	Gap Junction Protein Alpha 3	2.408
GJB2	Gap Junction Protein Beta 2	2.349
GJB6	Gap Junction Protein Beta 6	1.942
GPN1	GPN-Loop Gtpase 1	2.204
GPSM1	G Protein Signaling Modulator 1	1.954
GRAMD1C	GRAM Domain Containing 1C	2.383
HABP2	Hyaluronan Binding Protein 2	2.536
HAL	Histidine Ammonia-Lyase	1.817
HCFC1	Host Cell Factor C1	2.042
HDLBP	High Density Lipoprotein Binding Protein	2.457
HECTD1	HECT Domain E3 Ubiquitin Protein Ligase 1	2.577
HHATL	Hedgehog Acyltransferase Like	2.233
HIPK2	Homeodomain Interacting Protein Kinase 2	1.769
HIPK3	Homeodomain Interacting Protein Kinase 3	2.468
HNF4A	Hepatocyte Nuclear Factor 4 Alpha	2.430
HOXA11	Homeobox A11	1.781
HOXA7	Homeobox A7	2.221
HOXB2	Homeobox B2	2.564
HOXB3	Homeobox B3	2.196
HOXB4	Homeobox B4	2.286
HOXB8	Homeobox B8	2.429
HOXC8	Homeobox C8	1.868
HOXD11	Homeobox D11	2.297
HP1BP3	Heterochromatin Protein 1 Binding Protein 3	2.468
HSPA8	Heat Shock Protein Family A (Hsp70) Member 8	2.311
HSP90AB1	Heat Shock Protein 90 Alpha Family Class B Member 1	2.077
ID2	Inhibitor Of DNA Binding 2	1.762
IGFBP1	Insulin Like Growth Factor Binding Protein 1	2.628
ILK	Integrin Linked Kinase	2.476
IQGAP3	IQ Motif Containing Gtpase Activating Protein 3	2.067
IRX1	Iroquois Homeobox 1	1.757
IRX3	Iroquois Homeobox 3	2.028
ITGA7	Integrin Subunit Alpha 7	1.797
JUNB	Junb Proto-Oncogene, AP-1 Transcription Factor Subunit	1.798
KLHL41	Kelch Like Family Member 41	2.226
KDM5C	Lysine Demethylase 5C	2.295
KHSRP	KH-Type Splicing Regulatory Protein	1.757

KLC1	Kinesin Light Chain 1	1.956
KLF2	Kruppel Like Factor 2	1.884
KLK10	Kallikrein Related Peptidase 10	1.803
KRT16	Keratin 16	2.362
LAMA2	Laminin Subunit Alpha 2	2.159
LDHA	Lactate Dehydrogenase A	2.445
LGALS9C	Galectin 9C	2.444<br>2.431
LMOD3	Leiomodin 3	2.253
MAFB	MAF Bzip Transcription Factor B	2.177<br>1.848
MARCH3	Membrane Associated Ring-CH-Type Finger 3	1.768
MARK2	Microtubule Affinity Regulating Kinase 2	1.886
MARK4	Microtubule Affinity Regulating Kinase 4	2.048
MAT2A	Methionine Adenosyltransferase 2A	2.331
MATN4	Matrilin 4	2.010
MEIS3	Meis Homeobox 3	1.797
MIA3	MIA Family Member 3, ER Export Factor	2.299
MOSPD3	Motile Sperm Domain Containing 3	1.794
MPRIP	Myosin Phosphatase Rho Interacting Protein	1.856
MSGN1	Mesogenin 1	2.320
MTA2	Metastasis Associated 1 Family Member 2	2.213
MUC1	Mucin 1, Cell Surface Associated	2.164
MUC2	Mucin 2, Oligomeric Mucus/Gel-Forming	1.943
MVP	Major Vault Protein	1.970
MXI1	MAX Interactor 1, Dimerization Protein	1.998
MYF5	Myogenic Factor 5	1.854
MYH10	Myosin Heavy Chain 10	2.374
MYH3	Myosin Heavy Chain 3	1.814
MYO1E	Myosin IE	2.100
MYOC	Myocilin	2.270
MYOD1	Myogenic Differentiation 1	2.114
MYOT	Myotilin	1.863
MYOZ2	Myozenin 2	2.157
NAV2	Neuron Navigator 2	2.374
NEFM	Neurofilament Medium	1.880
NEXN	Nexilin F-Actin Binding Protein	2.605
NR4A1	Nuclear Receptor Subfamily 4 Group A Member 1	1.983<br>1.935
NT5C3A	5'-Nucleotidase, Cytosolic IIIA	2.231
ODAM	Odontogenic, Ameloblast Associated	2.189
OGDH	Oxoglutarate Dehydrogenase	2.271
OTUB1	OTU Deubiquitinase, Ubiquitin Aldehyde Binding 1	1.825
PAN3	PAN3 Poly(A) Specific Ribonuclease Subunit	1.838
PAX7	Paired Box 7	2.107
PBRM1	Polybromo 1	1.808
PCK1	Phosphoenolpyruvate Carboxykinase 1	2.415
PCOLCE	Procollagen C-Endopeptidase Enhancer	1.891
PCP4L1	Purkinje Cell Protein 4 Like 1	1.760
PDLIM7	PDZ And LIM Domain 7	2.406
PFN1	Profilin 1	1.830
PI4KB	Phosphatidylinositol 4-Kinase Beta	2.040
PITX1	Paired Like Homeodomain 1	1.982
PLCL2	Phospholipase C Like 2	1.910

PLEC	Plectin	1.910
PMM2	Phosphomannomutase 2	2.219
PMPCA	Peptidase, Mitochondrial Processing Alpha Subunit	2.078
PNPLA7	Patatin Like Phospholipase Domain Containing 7	2.329
PPARG	Peroxisome Proliferator Activated Receptor Gamma	2.031
PPDPF	Pancreatic Progenitor Cell Differentiation And Proliferation Factor	1.951<br>1.861<br>1.764
PPIP5K2	Diphosphoinositol Pentakisphosphate Kinase 2	2.365
PPP1R3C	Protein Phosphatase 1 Regulatory Subunit 3C	1.920
PPP2CA	Protein Phosphatase 2 Catalytic Subunit Alpha	1.752
PRKAG3	Protein Kinase AMP-Activated Non-Catalytic Subunit Gamma 3	2.264
PRKAR1A	Protein Kinase Camp-Dependent Type I Regulatory Subunit Alpha	2.358
PRRC2A	Proline Rich Coiled-Coil 2A	2.007
PRRC2B	Proline Rich Coiled-Coil 2B	2.629<br>1.766
PRTN3	Proteinase 3	2.164
PTGES3L	Prostaglandin E Synthase 3 Like	1.850
PTK2	Protein Tyrosine Kinase 2	1.933
PTPN1	Protein Tyrosine Phosphatase, Non-Receptor Type 1	2.050
PUS3	Pseudouridylate Synthase 3	2.044
PYGO2	Pygopus Family PHD Finger 2	2.199
QTRT2	Queuine Trna-Ribosyltransferase Accessory Subunit 2	2.063
R3HDM2	R3H Domain Containing 2	2.254
RAB10	RAB10, Member RAS Oncogene Family	2.092
RAB14	RAB14, Member RAS Oncogene Family	2.454
RAB1A	RAB1A, Member RAS Oncogene Family	2.078
RAB2A	RAB2A, Member RAS Oncogene Family	1.868
RAB40C	RAB40C, Member RAS Oncogene Family	1.954
RAP1GAP	RAP1 Gtpase Activating Protein	1.827
RAPSN	Receptor Associated Protein Of The Synapse	1.834
RBFOX3	RNA Binding Fox-1 Homolog 3	1.861
RBPMS	RNA Binding Protein With Multiple Splicing	1.847
RBPMS2	RNA Binding Protein With Multiple Splicing 2	2.039
RCSD1	RCSD Domain Containing 1	1.895
RDH11	Retinol Dehydrogenase 11 (All-Trans/9-Cis/11-Cis)	1.939
RFESD	Rieske Fe-S Domain Containing	2.189
RHOG	Ras Homolog Family Member G	1.754
RIPPLY3	Ripply Transcriptional Repressor 3	1.763
SCN2A	Sodium Voltage-Gated Channel Alpha Subunit 2	1.962
SCN3A	Sodium Voltage-Gated Channel Alpha Subunit 3	1.897
SDS	Serine Dehydratase	2.156
SEC14L1	SEC14 Like Lipid Binding 1	1.956
SELENOP	Selenoprotein P	2.580
SENP3	SUMO1/Sentrin/SMT3 Specific Peptidase 3	2.224<br>2.096
SEPP1	Selenoprotein P	2.580
SERPIND1	Serpin Family D Member 1	1.799
SETDB1	SET Domain Bifurcated 1	2.121<br>2.078
SGCB	Sarcoglycan Beta	2.636
SH3BGR	SH3 Domain Binding Glutamate Rich Protein	2.590
SHC1	SHC Adaptor Protein 1	2.159

SIK1	Salt Inducible Kinase 1	2.081
SIX1	SIX Homeobox 1	2.351
SLC16A3	Solute Carrier Family 16 Member 3	1.815
SLC26A6	Solute Carrier Family 26 Member 6	2.413
SMC3	Structural Maintenance Of Chromosomes 3	2.244
SMURF1	SMAD Specific E3 Ubiquitin Protein Ligase 1	1.824
SOD3	Superoxide Dismutase 3	2.046
SOX10	SRY-Box 10	1.881
SP3	Sp3 Transcription Factor	2.582
SRF	Serum Response Factor	1.948
SSBP1	Single Stranded DNA Binding Protein 1	2.156
STAT5A	Signal Transducer And Activator Of Transcription 5A	1.959
SYNPO2L	Synaptopodin 2 Like	2.326
SYPL2	Synaptophysin Like 2	2.069
TACR1	Tachykinin Receptor 1	2.165
TAL1	TAL Bhlh Transcription Factor 1, Erythroid Differentiation Factor	2.210
THBS4	Thrombospondin 4	2.598
THOC6	THO Complex 6	2.413
TJAP1	Tight Junction Associated Protein 1	1.855<br>1.751
TLX3	T Cell Leukemia Homeobox 3	2.583
TMEM163	Transmembrane Protein 163	1.757
TMEM72	Transmembrane Protein 72	1.785
TOP2B	DNA Topoisomerase II Beta	1.882
TRAK1	Trafficking Kinesin Protein 1	2.113<br>2.038
TRAPPC10	Trafficking Protein Particle Complex 10	2.088
TRAPPC9	Trafficking Protein Particle Complex 9	1.821
TRIM55	Tripartite Motif Containing 55	2.627
TRIM66	Tripartite Motif Containing 66	1.763
TSPAN18	Tetraspanin 18	2.170
TUBA8	Tubulin Alpha 8	1.925
TXLNB	Taxilin Beta	2.135
UBA1	Ubiquitin Like Modifier Activating Enzyme 1	1.991
UBAP2L	Ubiquitin Associated Protein 2 Like	2.037
UBE2R2	Ubiquitin Conjugating Enzyme E2 R2	1.786
UBE2V1	Ubiquitin Conjugating Enzyme E2 V1	1.787
UBQLN1	Ubiquilin 1	2.288
UBQLN4	Ubiquilin 4	2.044
UBR5	Ubiquitin Protein Ligase E3 Component N-Recognin 5	2.022
UCMA	Upper Zone Of Growth Plate And Cartilage Matrix Associated	1.756
UPK1B	Uroplakin 1B	1.934
VAMP2	Vesicle Associated Membrane Protein 2	2.585
VLDLR	Very Low Density Lipoprotein Receptor	1.799
WASHC1	WASH Complex Subunit 1	1.772
WBP2	WW Domain Binding Protein 2	1.891
WDR54	WD Repeat Domain 54	1.952
WEE1	WEE1 G2 Checkpoint Kinase	2.349
XIRP1	Xin Actin Binding Repeat Containing 1	1.770
XPO1	Exportin 1	1.953
ZNF652	Zinc Finger Protein 652	1.791
ZNF653	Zinc Finger Protein 653	1.795
ZNF703	Zinc Finger Protein 703	2.087

ZYX	Zyxin	2.103<br>1.891

B.

HGNC	Gene Name	Log2 Fold Change
	DECREASED	
ADAMTS13	Adam Metallopeptidase With Thrombospondin Type 1 Motif 13	-0.499
ADGRG2	Adhesion G Protein-Coupled Receptor G2	-0.573
AKAP12	A-Kinase Anchoring Protein 12	-0.502<br>-0.618
ALDH1A2	Aldehyde Dehydrogenase 1 Family Member A2	-0.413<br>-0.493
ALX1	Alx Homeobox 1	-0.431<br>-0.586
ALX4	Alx Homeobox 4	-0.733<br>-0.926
ATP13A4	Atpase 13A4	-0.516
BGN	Biglycan	-0.675
BMP2	Bone Morphogenetic Protein 2	-0.456
BMP5	Bone Morphogenetic Protein 5	-0.696
CLEC11A	C-Type Lectin Domain Containing 11a	-0.625
CLU	Clusterin	-0.447
COCH	Cochlin	-0.950
COL9A1	Collagen Type IX Alpha 1 Chain	-0.465<br>-0.583
COL9A2	Collagen Type IX Alpha 2 Chain	-0.345
COL9A3	Collagen Type IX Alpha 3 Chain	-0.374
COLEC12	Collectin Subfamily Member 12	-0.420<br>-0.423
CORIN	Corin, Serine Peptidase	-0.460
CPN1	Carboxypeptidase N Subunit 1	-0.537
CPXM1	Carboxypeptidase X, M14 Family Member 1	-0.429
CRABP2	Cellular Retinoic Acid Binding Protein 2	-0.485<br>-0.939
CYP26A1	Cytochrome P450 Family 26 Subfamily A Member 1	-0.690<br>-1.637
CYP26B1	Cytochrome P450 Family 26 Subfamily B Member 1	-0.697
CYP26C1	Cytochrome P450 Family 26 Subfamily C Member 1	-0.440
DHRS3	Dehydrogenase/Reductase 3	-0.734<br>-1.052
DMBX1	Diencephalon/Mesencephalon Homeobox 1	-0.648
DNAJC12	Dnaj Heat Shock Protein Family (Hsp40) Member C12	-0.659
EDNRB	Endothelin Receptor Type B	-0.474
EMILIN1	Elastin Microfibril Interfacer 1	-0.356
EN2	Engrailed Homeobox 2	-0.618
ENTPD1	Ectonucleoside Triphosphate Diphosphohydrolase 1	-0.497
EYA2	Eya Transcriptional Coactivator And Phosphatase 2	-0.475
F2R	Coagulation Factor Ii Thrombin Receptor	-0.465
FAM151A	Family With Sequence Similarity 151 Member A	-0.417

FOXC1	Forkhead Box C1	-0.508
FOXD1	Forkhead Box D1	-0.501
FOXI4	Forkhead Box I4	-0.563
FOXP4	Forkhead Box P4	-0.425
GATA2	GATA Binding Protein 2	-0.587<br>-0.815
GATA3	GATA Binding Protein 3	-0.476<br>-0.668
GH1	Growth Hormone 1	-0.484<br>-0.660
GLIS3	GLIS Family Zinc Finger 3	-0.536
HAS2	Hyaluronan Synthase 2	-0.526<br>-0.710
HEBP2	Heme Binding Protein 2	-0.485
HIC1	HIC ZBTB Transcriptional Repressor 1	-0.774
HMGA2	High Mobility Group AT-Hook 2	-0.485
HOXA4	Homeobox A4	-0.562
HSPA12B	Heat Shock Protein Family A (Hsp70) Member 12B	-0.373
IL11RA	Interleukin 11 Receptor Subunit Alpha	-0.407
IL13RA2	Interleukin 13 Receptor Subunit Alpha 2	-0.318
KIAA1755	KIAA1755	-0.435
KRT19	Keratin 19	-0.617
MATN4	Matrilin 4	-0.518
MYADM	Myeloid Associated Differentiation Marker	-0.467
MYL4	Myosin Light Chain 4	-0.495
MYOM3	Myomesin 3	-0.394
NEFM	Neurofilament Medium	-0.474
PAH	Phenylalanine Hydroxylase	-0.575
PRRT3	Proline Rich Transmembrane Protein 3	-0.547
QDPR	Quinoid Dihydropteridine Reductase	-0.446
RARB	Retinoic Acid Receptor Beta	-0.731
RARG	Retinoic Acid Receptor Gamma	-0.375
RHBG	Rh Family B Glycoprotein (Gene/Pseudogene)	-0.659<br>-0.440
S100A9	S100 Calcium Binding Protein A9	-0.474
SEPT9	Septin 9	-0.376
SFRP1	Secreted Frizzled Related Protein 1	-0.498<br>-0.697
SIX2	SIX Homeobox 2	-0.516<br>-0.805
SNCG	Synuclein Gamma	-0.466
SSPO	SCO-Spondin	-0.542
SSTR2	Somatostatin Receptor 2	-0.717
TAL2	TAL Bhlh Transcription Factor 2	-0.542
TGFBI	Transforming Growth Factor Beta Induced	-0.709
TRABD2B	TRAB Domain Containing 2B	-0.411
UCMA	Upper Zone Of Growth Plate And Cartilage Matrix Associated	-0.578
WNT1	Wnt Family Member 1	-0.347
INCREASED		
AIFM3	Apoptosis Inducing Factor, Mitochondria Associated 3	0.406
ATOH7	Atonal Bhlh Transcription Factor 7	0.402
CAPN3	Calpain 3	0.570
CHODL	Chondrolectin	0.538

CLUL1	Clusterin Like 1	0.354
CRYBG1	Crystallin Beta-Gamma Domain Containing 1	0.393
DLX2	Distal-Less Homeobox 2	0.527
ERMIN	Ermin	0.386
FRZB	Frizzled Related Protein	0.598
HBD	Hemoglobin Subunit Delta	0.590
HEMGN	Hemogen	0.452
IGFBP2	Insulin Like Growth Factor Binding Protein 2	0.407
LHX1	LIM Homeobox 1	0.414
PCK2	Phosphoenolpyruvate Carboxykinase 2, Mitochondrial	0.469
SIM2	Single-Minded Family bHLH Transcription Factor 2	0.538<br>0.463
SLC39A8	Solute Carrier Family 39 Member 8	0.542
SMOC1	SPARC Related Modular Calcium Binding 1	0.541
VSX1	Visual System Homeobox 1	0.585
